# Assessment of Novel Mesothelin-Specific Human Antibody Domain VH-Fc Fusion Proteins-Based PET Agents

**DOI:** 10.1021/acsomega.3c04492

**Published:** 2023-11-08

**Authors:** Zehua Sun, Ambika P. Jaswal, Xiaojie Chu, Harikrishnan Rajkumar, Angel G. Cortez, Robert Edinger, Max Rose, Anders Josefsson, Abhinav Bhise, Ziyu Huang, Rieko Ishima, John W Mellors, Dimiter S. Dimitrov, Wei Li, Jessie R. Nedrow

**Affiliations:** † Center for Antibody Therapeutics, Division of Infectious Diseases, Department of Medicine, University of Pittsburgh School of Medicine, Pittsburgh, Pennsylvania 15261, United States; ‡ Department of Neurological Surgery, University of Pittsburgh School of Medicine, Pittsburgh, Pennsylvania 15261, United States; § Hillman Cancer Center, University of Pittsburgh School of Medicine, Pittsburgh, Pennsylvania 15261, United States; ¶ Department of Structural Biology, University of Pittsburgh School of Medicine, Pittsburgh, Pennsylvania 15261, United States; # Department of Radiology, University of Pittsburgh School of Medicine, Pittsburgh, Pennsylvania 15261, United States

## Abstract

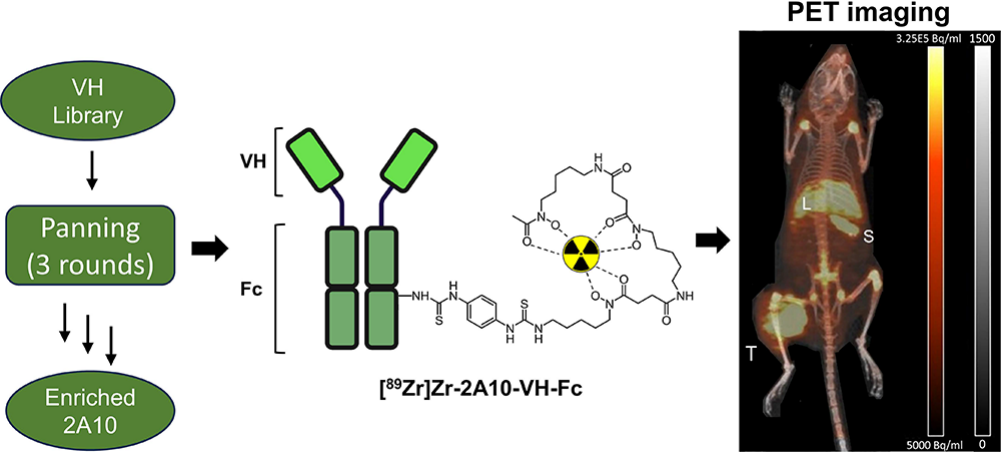

Mesothelin (MSLN) is a tumor-associated antigen found in a variety of cancers and is a target for imaging and therapeutic applications in MSLN-expressing tumors. We have developed high affinity anti-MSLN human VH domain antibodies, providing alternative targeting vectors to conventional IgG antibodies that are associated with long-circulating half-lives and poor penetration of tumors, limiting antitumor activity in clinical trials. Based on two newly identified anti-MSLN VH binders (3C9, 2A10), we generated VH-Fc fusion proteins and modified them for zirconium-89 radiolabeling to create anti-MSLN VH-Fc PET tracers. The focus of this study was to assess the ability of PET-imaging to compare the *in vivo* performance of anti-MSLN VH-Fc fusion proteins (2A10, 3C9) targeting different epitopes of MSLN vs IgG1 (m912; a clinical benchmark antibody with an overlapped epitope as 2A10) for PET imaging in a mouse model of colorectal cancer (CRC). The anti-MSLN VH-Fc fusion proteins were successfully modified and radiolabeled with zirconium-89. The resulting MSLN-targeted PET-imaging agents demonstrated specific uptake in the MSLN-expressing HCT116 tumors. The *in vivo* performance of the MSLN-targeted PET-imaging agents utilizing VH-Fc showed more rapid and greater accumulation and deeper penetration within the tumor than the full-length IgG1 m912-based PET-imaging agent. Furthermore, PET imaging allowed us to compare the pharmacokinetics of epitope-specific VH domain-based PET tracers. Overall, these data are encouraging for the incorporation of PET imaging to assess modified VH domain structures to develop novel anti-MSLN VH domain-based therapeutics in MSLN-positive cancers as well as their companion PET imaging agents.

## Introduction

Mesothelin (MSLN) is a glycosyl phosphatidyl inositol (GPI)-anchored protein that was first described in human ovarian carcinoma cells.^1^ MSLN expression is limited in normal tissues with expression found only in the mesothelia and sparse expression in the trachea, tonsil, and fallopian tube.^1,2^ However, it is overexpressed by a variety of solid tumors, including mesothelioma, colorectal, pancreatic, lung, and ovarian cancers.^3^ In addition, it has been shown that patients’ prognoses are worse when diffused MSLN expression is found, and these patients have a decrease in overall survival when tumors overexpress MSLN.^4,5^ MSLN’s overexpression in cancers and limited expression in normal tissues have made this biomarker desirable for targeted therapies.

Antibody-dependent therapies targeting MSLN, including antibody drug conjugates (ADC), are being evaluated in clinical trials; however, they have had minimal improvements in therapeutic outcomes.^6−10^ Intact IgG antibodies have been widely utilized in anti-MSLN targeted therapy due to its high binding affinity and specificity.^11^ For example, our group has identified a full-length antibody, m912, which induced specific ADCC against MSLN overexpression cancer cells.^12^ m912 has been evaluated in phase I/II clinical trials in the context of CAR-T cell therapy and exhibited significant clinical efficacy.^13^ However, a full-length antibody typically encounters obstructions for penetrating solid tumors due to large molecular size, i.e., large hydrodynamic radius leading to low diffusion coefficient in tumor/stromal interstitial.^14^ These hurdles may limit applications of fully intact antibodies as anti-MSLN targeted therapies. The development and characterization of novel anti-MSLN agents with high affinity, specificity, and a smaller molecular size have the potential to overcome the challenges observed in clinical trials of antibody-dependent therapies for MSLN-expressing cancers.

The development of novel targeting agents for anti-MSLN therapies will help to improve their therapeutic effectiveness. Antibody domains have advantages over traditional full-length antibodies, including increased tumor penetration, customized molecular formats, and compatible pharmacokinetics (PK) for therapy. Previously, we generated a large fully human VH domain phage-displayed library.^15−17^ From this library we were able to isolate a variety of human immunoglobulin variable heavy chain (VH) domains that target SARS-CoV-2, CD22, and PD-L1 as well as MSLN. An anti-MSLN VH domain 3C9, modified as a VH-Fc fusion protein drug conjugate, showed promising therapeutic results in MSLN-expressing xenograft tumors.^18^ However, the current tools to assess and optimize novel targeting agents for anti-MSLN therapies are limited. In the present study, we aim to utilize PET-imaging to assess and compare anti-MSLN targeting agents, including a VH-Fc fusion protein utilizing a newly identified MSLN specific human antibody domain, 2A10; our previously developed VH-Fc 3C9 fusion protein; and a fully intact IgG1 (m912; a clinical benchmark antibody with an overlapped epitope as 2A10).

## Results

### Generation and Evaluation of the 2A10 VH Domain and VH-Fc Fusion Proteins

**Figure 1:**
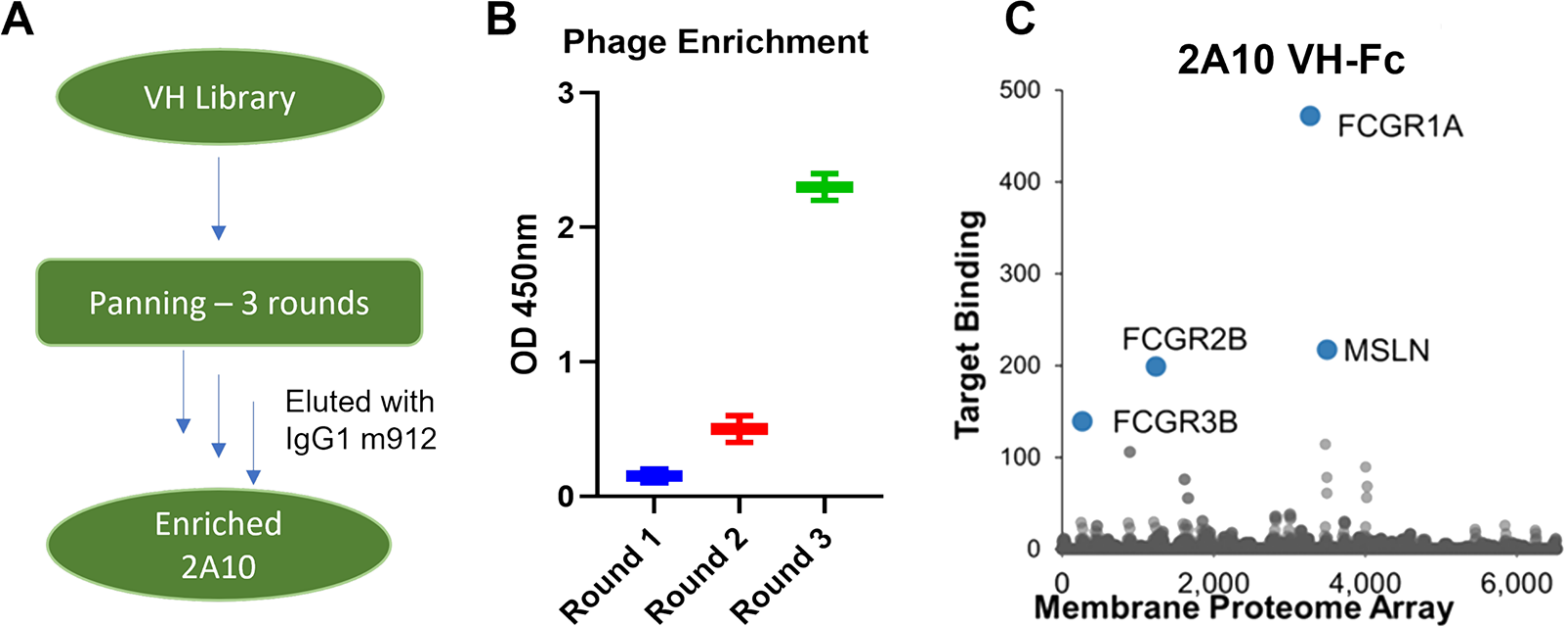
(A) Schematic of the isolation and (B) enrichment of the anti-MSLN VH domain 2A10. (C) Membrane proteome array of 2A10 VHFc.

The 2A10 VH domain was screened from a phage displayed library panning with competitive elution by IgG1 m912 and was significantly enriched with three rounds of panning (Figure 1A,B). A membrane proteome array (MPA) platform was used to test specificities of VH-Fc 2A10 against a total 6,000 different human membrane proteins in a high-throughput screening manner based on flow cytometry.^19^ Potential targets showing signals include MSLN, FcγRs (Ia, IIB, IIIB), SIA7F (gene name ST6GALNAC6), solute carrier family 25 member (gene name 35SLC25A35), and CD325 (gene name CDH2) according to the signal potency (Figure 1C). Potential targets showing signal were further verified by flow cytometry. VH-Fc 2A10 demonstrated binding to MSLN as well as to the Fc receptors (FcγRs). The FcγRs (Ia, IIB, IIIB) are associated with low affinity binding to the Fc portion of IgG1s.^20^ VH-Fc 2A10 showed no binding to SIA7F, solute carrier family 25 member, or CD325.

The ELISA assay demonstrated that the 2A10 VH domain exhibited an EC_50_ around 10 ± 3 nmol as compared to m912 (70 ± 3 nmol) and 3C9 VH (0.3 ± 0.1 nmol) (Supporting Information (SI) Figure 1A). The addition of the anti-MSLN antibody, m912 IgG1, increased the EC_50_ value to 70 nM, indicating that the 2A10 VH domain competes with the m912 IgG1 for binding (SI Figure 1B). However, the addition of the 3C9-VH-Fc^16^ did not impact the binding of 2A10 VH, indicating that they target nonoverlapping epitopes (SI Figure 1C). SPR analysis demonstrated that the 2A10 VH domain and VH-Fc fusion protein had high affinities to human MSLN, with a K_D_ of 2.4 ± 0.01 nmol and 2.0 ± 0.1 nmol (SI Figure 2), which were slightly lower than those of the 3C9 VH and VH-Fc fusion proteins as reported previously, 2.6 and 7.4 nmol, respectively.^16^ The K_D_ of 2A10 VH-Fc under SPR conditions more suitable to VH-Fc decreased to 0.6 ± 0.01 nmol. The VH-Fc fusion proteins did not demonstrate cross-reactivity to murine MSLN as compared to the m912 IgG1 (positive control) that has previous demonstrated cross-reactivity between human and murine MSLN (SI Figure 5).^12^ The propensity for aggregation of the 2A10 VH domain and VH-Fc fusion protein was evaluated by SEC (SI Figure 3). Low aggregation of both the 2A10 VH domain (3%) and VH-Fc protein fusion (<1%) was observed, which is consistent with the anti-MSLN 3C9 VH domain and VH-Fc fusion protein.^16^

### Conjugation and Radiolabeling of Anti-MSLN Antibody and VH-Fc Fusion Protein

The ratios of DFO chelators to VH-Fc fusion proteins are as follows: 1.37 ± 0.17 (2A10), 1.52 ± 0.18 (3C9), and 1.07 ± 0.40 (Ab6). The fully intact IgG1 m912 ratio was slightly lower at 0.80 ± 0.23. Assessment of the DFO conjugates by SPR demonstrated that the *K*_D_ for the anti-MSLN VH-Fc (3C9, 2A10) and IgG1-m912 conjugates had high affinity for human MSLN: 29.7 ± 17.1, 25.7 ± 13.9, and 5.69 ± 1.71 nmol, respectively (SI Figure 4). The untargeted VH-Fc Ab6 conjugate’s *K*_D_ was not determinable, as expected.

Radiolabeling yields ranged between 89.1 and 98.5%: 95.2% [^89^Zr]Zr-2A10, 89.1% [^89^Zr]Zr-3C9, and 98.5% [^89^Zr]Zr-Ab6 as well as 89.2% for [^89^Zr]Zr-m912. All radioconjugates were purified and/or buffered exchanged with PBS to obtain a RLP of >95%. The molar activities for the VH-Fc fusion proteins ranged between 1.08 and 1.50 MBq/μmol, and the molar activity for the m912 IgG1 antibody was 1.65–2.06 MBq/μmol (Table 1 and SI Table 1).

**Table 1:** Summary of [^89^Zr]Zr-Labeled Radiotracers Injected into HCT116-Tumor Bearing Mice

	**activity injected**				
**[**^**89**^**Zr]Zr-labeled PET tracer**	**MBq**	**μCi**	**protein amount**(μg/100 μL)	**molar activity**(MBq/μmol)	**Number of Mice Injected**
**Ab6-VH-Fc**	1.55–1.81	42–49	16	1.08 ± 0.02	4
**2A10-VH-Fc**	1.89–2.26	51–61	19	1.14 ± 0.09	3
**3C9-VH-Fc**	1.77–1.89	48–51	18	1.24 ± 0.04	4
**M912-IgG1**	1.19–1.24	32–33	16	1.65 ± 0.03	4

*In vivo* stabilities for [^89^Zr]Zr-2A10, [^89^Zr]Zr-3C9, and [^89^Zr]Zr-m912 were monitored at 24 and 48 h. At 24 h, [^89^Zr]Zr-2A10 and [^89^Zr]Zr-3C9 demonstrated ≥90% stability as compared to ∼85% for [^89^Zr]Zr-m912 (see SI Table 2). At 48 h, all radioconjugates were ≥85% intact.

### MSLN Expression in HCT116 Cells and the Tumor Model

**Figure 2:**
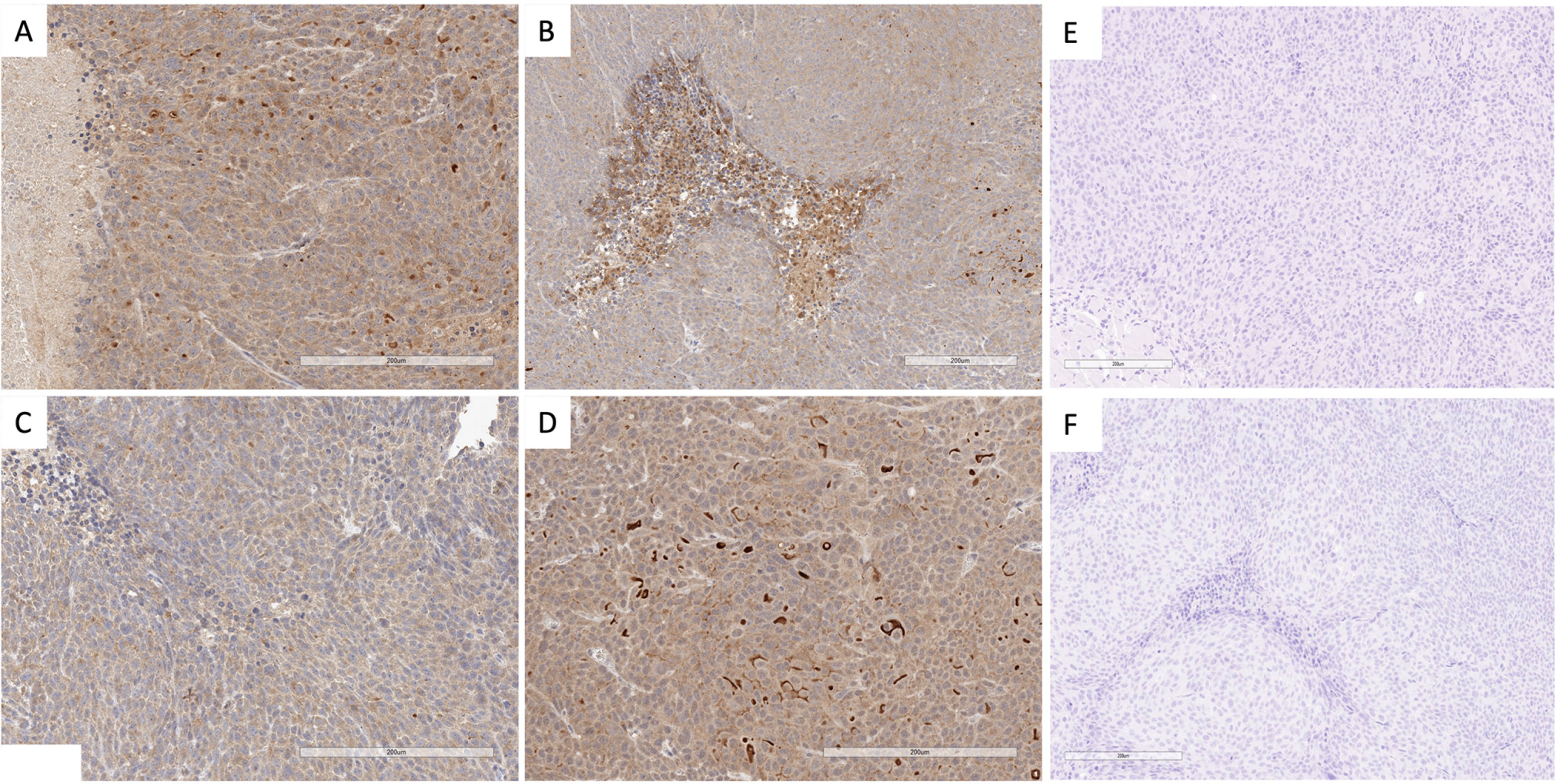
Representative immunohistochemistry of mesothelin (MSLN) expression in sections of HCT116 xenograft tumors (A–D). Nonspecific staining was assessed using an isotype control primary antibody (E, F).

MSLN expression in HCT116 cells was confirmed by western blot analysis (SI Figure 6). MSLN-specific IHC staining was demonstrated in HCT116 xenograft tumors having heterogeneous expression (Figure 2). MSLN staining was not observed in the following murine tissues: spleen, kidney, liver, marrow, and bone (SI Figure 7).

### PET Imaging Studies

**Figure 3:**
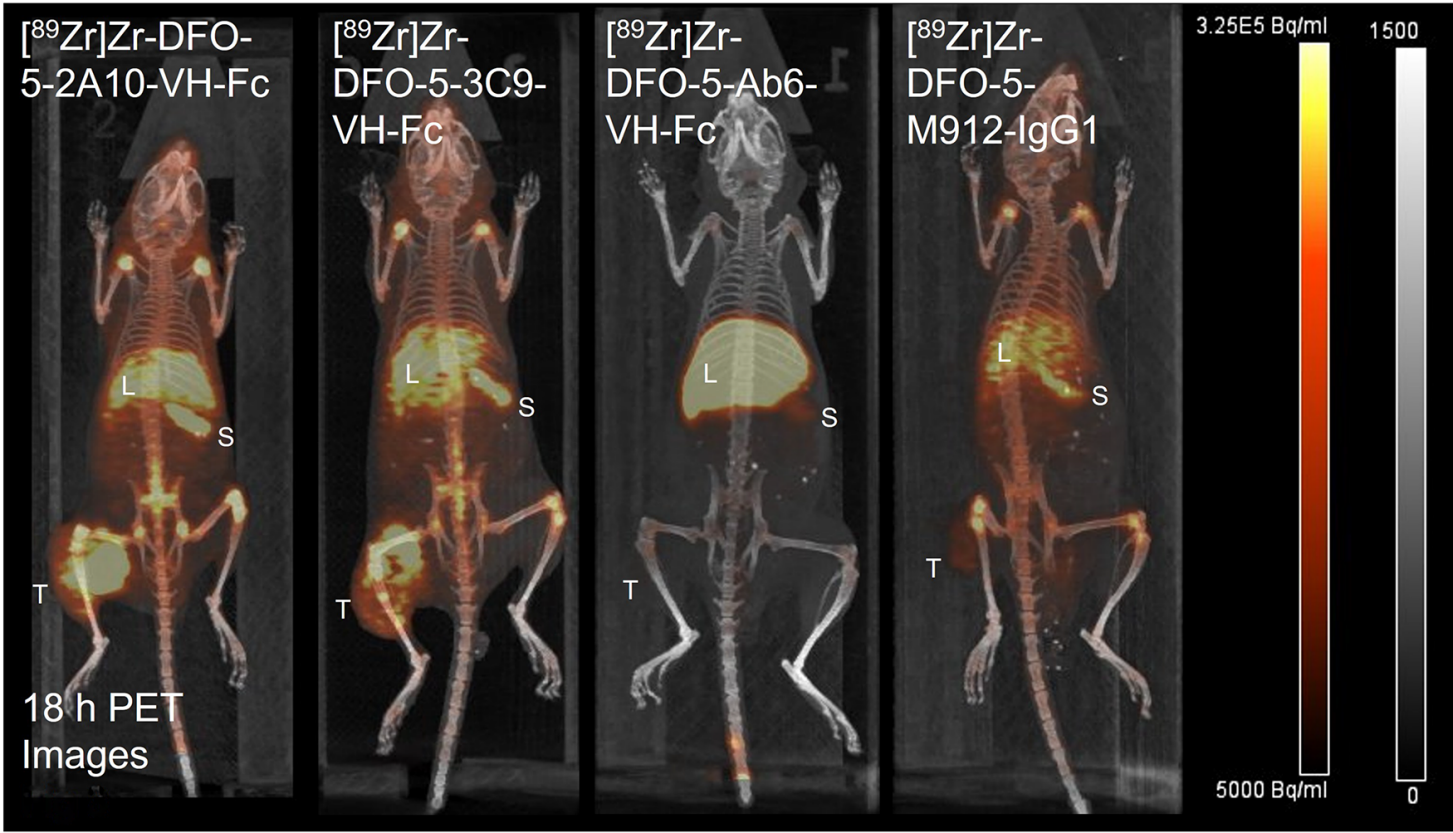
PET-imaging of NCG mice bearing HCT116 tumors approximately 18 h p.i. of the following zirconium-89 labeled agents: 2A10-VHFc (2.3 MBq[61 μCi]/16 μg), 3C9-VHFc (2.1 MBq[57 μCi]/19 μg), Ab6-VHFc (1.8 MBq[49 μCi]/16 μg), and M912-IgG (1.7 MBq[45 μCi]/18 μg). Denotations: L – iver, S – Spleen, and T – HCT116-tumor.

**Figure 4:**
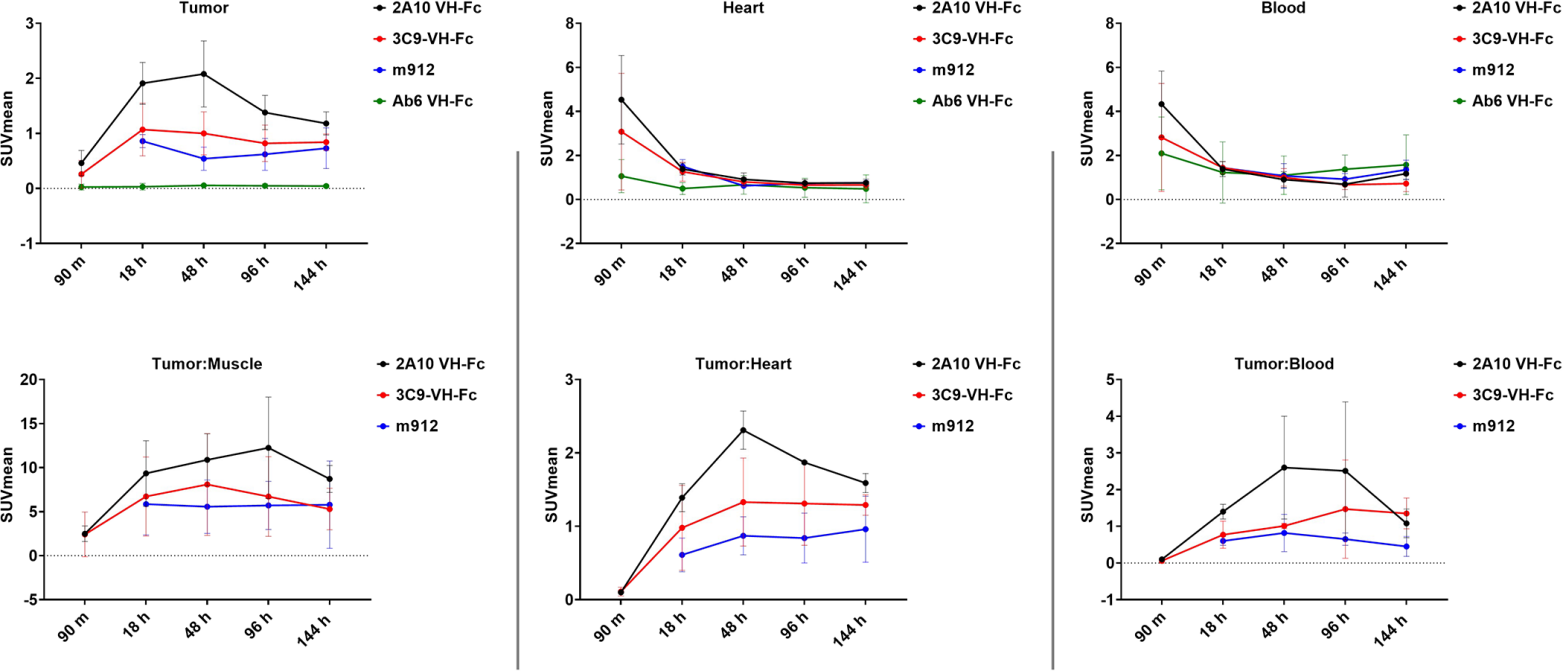
SUV_mean_ of zirconium-89 labeled compounds over a 6 day window.

PET imaging studies were carried out in NCG mice (8–10 weeks) bearing HCT116-tumors at 90 min (VH-Fc radioconjugates only); 18, 48, 96, and 144 h (Figure 3, Figure 4). The anti-MSLN VH-Fcs (2A10, 3C9) and IgG1 m912 demonstrated accumulation within the MSLN-positive HCT116 tumor, with the highest uptake occurring at 18 h for [^89^Zr]Zr-VH-Fc 3C9 (1.07 ± 0.48 SUV_mean_) and [^89^Zr]Zr-m912 (0.86 ± 0.12 SUV_mean_) followed by slight decreases over the 144 h window (Table 2, Figure 4). [^89^Zr]Zr-VH-Fc 2A10 achieved the highest accumulation at 48 h (2.08 ± 0.60 SUV_mean_), and consistently demonstrated significantly higher tumor uptake than [^89^Zr]Zr-m912 (*p* ≤ 0.05) at all time points (90 m not applicable) and [^89^Zr]Zr-VH-Fc 3C9 (*p* ≤ 0.001) at 18 and 48 h. All anti-MSLN PET agents demonstrated significantly higher tumor uptake (*p* ≤ 0.05) at 18, 48, and 96 h as compared to the negative control [^89^Zr]Zr- VH-Fc Ab6, while only the anti-MSLN VH-Fcs PET agents (2A10, 3C9) demonstrated significantly higher tumor uptake at 144 h (*p* ≤ 0.01). The anti-MSLN PET agents were further evaluated by comparing their SUV_mean_ ratios: tumor-to-muscle, tumor-to-heart, and tumor-to-blood. Significant differences were observed only for tumor-to-muscle ratios at 144 h comparing [^89^Zr]Zr-VH-Fc 2A10 and [^89^Zr]Zr-m912 (8.71 ± 1.53 vs 5.78 ± 4.96; p = 0.025). Tumor-to-heart ratios showed significantly higher SUVmeans of the anti-VH-Fc agents (2A10,3C9) as compared to [^89^Zr]Zr-m912 at 144 h (*p* ≤ 0.001). Tumor-to-blood demonstrated significantly higher ratios for [^89^Zr]Zr-VH-Fc 2A10 at 48 h as compared to both [^89^Zr]Zr-VH-Fc 3C9 (*p* ≤ 0.014) and [^89^Zr]Zr-m912 (*p* = 0.006). All the anti-MSLN PET agents as well as the negative control PET agent demonstrated signals in the liver, spleen, and bone/marrow. The liver accumulation is likely associated with catabolism of the radioconjugates, which is supported by the negative control, [^89^Zr]Zr- VH-Fc Ab6, showing signal in the liver. The PET-images of [^89^Zr]Zr-VH-Fc Ab6 showed a reduced signal in the spleen and joints (marrow) as compared to the anti-MSLN PET-agents; however, the signal is still present when scaled separately from anti-MSLN PET-tracers. PET imaging studies were repeated for [^89^Zr]Zr-VH-Fc 2A10 and the positive control [^89^Zr]Zr-m912 with and without excess unlabeled Fc block (irrelevant anti-SARS-CoV-2 IgG1 ab1) to demonstrate Fc-mediated binding at 90 min; 24, 48, and 120 h (time points based on microPET/CT availability). The presence of the Fc Block demonstrated decreases in the marrow SUV_mean_ (SI Table 3, SI Figure 8,9); [^89^Zr]Zr-m912 marrow SUV_mean_ was significantly reduced at 24, 48, and 120 h (*p* ≤ 0.01) and [^89^Zr]Zr-VH-Fc 2A10 had a significant decrease at 48 h (p = 0.031) in the presence of the Fc block. [^89^Zr]Zr-VH-Fc 2A10 and [^89^Zr]Zr-m912’s tumor SUV_mean_ in the presence of the Fc block improved over the 120-h window as compared to the agents without block; significant increase in the tumor SUV_mean_ for both agents plus Fc Block was observed at 120 h ((*p* ≤ 0.01); SI Figure 8). The SUV_mean_ in the blood was significantly higher for both the [^89^Zr]Zr-VH-Fc 2A10 and [^89^Zr]Zr-m912 in the presence of the Fc block at 24 h (*p* ≤ 0.05) while the SUV_mean_ in the heart was significantly higher for both agents plus Fc block at 24 and 48 h (*p* ≤ 0.05) as well as at 120 h for [^89^Zr]Zr-m912.Tumor-to-blood ratios increased over the 120-h window for both agents with Fc block, demonstrating significantly higher ratios at 120 h (*p* ≤ 0.05). Tumor-to-heart ratios were higher for the [^89^Zr]Zr-VH-Fc 2A10 and [^89^Zr]Zr-m912 (except 120 h) without block; significance was observed at 24 and 48 h for [^89^Zr]Zr-VH-Fc 2A10. Tumor-to-muscle ratios increased for both [^89^Zr]Zr-VH-Fc 2A10 and [^89^Zr]Zr-m912 over the 120 h window in the presence of Fc Block, but without significance.

**Table 2:** SUV_mean_ Values for Anti-MSLN PET Tracers

[^89^Zr]Zr-DFO-5–2A10-VH-Fc (*n* = 3)		tumor	heart	blood	muscle	tumor: muscle	tumor: heart	tumor:blood
	90 m	0.46 ± 0.23	4.53 ± 2.01	4.33 ± 1.50	0.18 ± 0.09	2.48 ± 0.89	0.10 ± 0.01	0.10 ± 0.03
	18 h	1.91 ± 0.38	1.38 ± 0.27	1.38 ± 0.34	0.24 ± 0.12	9.34 ± 3.71	1.39 ± 0.19	1.40 ± 0.20
	48 h	2.08 ± 0.60	0.91 ± 0.29	0.90 ± 0.36	0.21 ± 0.11	10.88 ± 2.95	2.31 ± 0.26	2.60 ± 1.40
	96 h	1.38 ± 0.31	0.74 ± 0.16	0.69 ± 0.58	0.13 ± 0.05	12.25 ± 5.76	1.87 ± 0.02	2.51 ± 1.88
	144 h	1.18 ± 0.21	0.75 ± 0.17	1.17 ± 0.28	0.14 ± 0.05	8.71 ± 1.53	1.59 ± 0.13	1.08 ± 0.39

[^89^Zr]Zr-DFO-5–3C9- VH-Fc (*n* = 4)		tumor	heart	blood	muscle	tumor: muscle	tumor: heart	tumor: blood
	90 m	0.26 ± 0.25	3.08 ± 2.65	2.82 ± 2.45	0.12 ± 0.03	2.41 ± 2.54	0.11 ± 0.06	0.06 ± 0.07
	18 h	1.07 ± 0.48	1.26 ± 0.43	1.44 ± 0.28	0.20 ± 0.08	6.72 ± 4.49	0.98 ± 0.58	0.77 ± 0.37
	48 h	1.00 ± 0.39	0.80 ± 0.20	1.00 ± 0.40	0.16 ± 0.07	8.08 ± 5.78	1.33 ± 0.60	1.01 ± 0.07
	96 h	0.82 ± 0.33	0.65 ± 0.14	0.67 ± 0.22	0.15 ± 0.05	6.71 ± 4.52	1.31 ± 0.57	1.47 ± 1.34
	144 h	0.84 ± 0.15	0.66 ± 0.18	0.72 ± 0.37	0.18 ± 0.06	5.29 ± 2.36	1.29 ± 0.14	1.35 ± 0.42

[^89^Zr]Zr-DFO-5-m912 IgG1(*n* = 4)		tumor	heart	blood	muscle	tumor: muscle	tumor: heart	tumor: blood
	90 m							
	18 h	0.86 ± 0.12	1.50 ± 0.31	1.44 ± 0.11	0.22 ± 0.17	5.85 ± 3.51	0.61 ± 0.23	0.60 ± 0.12
	48 h	0.54 ± 0.21	0.62 ± 0.09	1.07 ± 0.56	0.12 ± 0.05	5.56 ± 3.03	0.87 ± 0.26	0.82 ± 0.51
	96 h	0.62 ± 0.29	0.73 ± 0.07	0.92 ± 0.26	0.11 ± 0.02	5.70 ± 2.73	0.84 ± 0.34	0.65 ± 0.17
	144 h	0.73 ± 0.37	0.76 ± 0.08	1.35 ± 0.43	0.18 ± 0.11	5.78 ± 4.96	0.96 ± 0.45	0.45 ± 0.27

[^89^Zr]Zr-DFO-5–3C9- VH-Fc (*n* = 4)		tumor	heart	blood	muscle	tumor: muscle	tumor: heart	tumor: blood
	90 m	0.26 ± 0.25	3.08 ± 2.65	2.82 ± 2.45	0.12 ± 0.03	2.41 ± 2.54	0.11 ± 0.06	0.06 ± 0.07
	18h	1.07 ± 0.48	1.26 ± 0.43	1.44 ± 0.28	0.20 ± 0.08	6.72 ± 4.49	0.98 ± 0.58	0.77 ± 0.37
	48 h	1.00 ± 0.39	0.80 ± 0.20	1.00 ± 0.40	0.16 ± 0.07	8.08 ± 5.78	1.33 ± 0.60	1.01 ± 0.07
	96 h	0.82 ± 0.33	0.65 ± 0.14	0.67 ± 0.22	0.15 ± 0.05	6.71 ± 4.52	1.31 ± 0.57	1.47 ± 1.34
	144 h	0.84 ± 0.15	0.66 ± 0.18	0.72 ± 0.37	0.18 ± 0.06	5.29 ± 2.36	1.29 ± 0.14	1.35 ± 0.42

[^89^Zr]Zr-DFO-5-m912 IgG1(*n* = 4)		tumor	heart	blood	muscle	tumor: muscle	tumor: heart	tumor: blood
	90 m							
	18h	0.86 ± 0.12	1.50 ± 0.31	1.44 ± 0.11	0.22 ± 0.17	5.85 ± 3.51	0.61 ± 0.23	0.60 ± 0.12
	48 h	0.54 ± 0.21	0.62 ± 0.09	1.07 ± 0.56	0.12 ± 0.05	5.56 ± 3.03	0.87 ± 0.26	0.82 ± 0.51
	96 h	0.62 ± 0.29	0.73 ± 0.07	0.92 ± 0.26	0.11 ± 0.02	5.70 ± 2.73	0.84 ± 0.34	0.65 ± 0.17
	144 h	0.73 ± 0.37	0.76 ± 0.08	1.35 ± 0.43	0.18 ± 0.11	5.78 ± 4.96	0.96 ± 0.45	0.45 ± 0.27

### iQID Imaging

**Figure 5:**
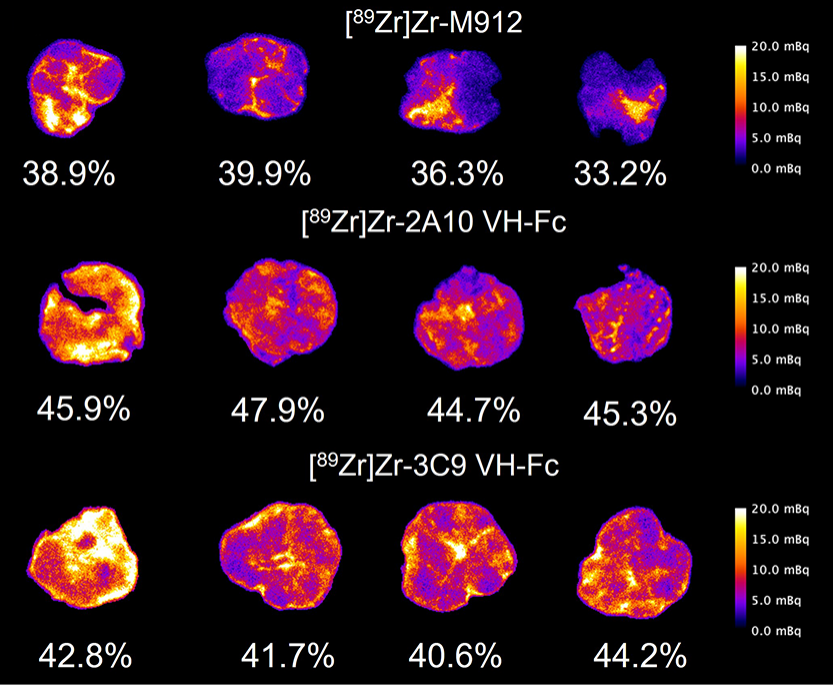
iQID-camera image of the activity distribution for zirconium-89 labeled m912, 2A10 VH-Fc, and 3C9 VH-Fc in HCT116-tumors. The distribution uniformities are displayed in individual sections. Note the activity scale bar has a range between 0 and 20 mBq but maximum activity is higher.

The microscale distribution of the [^89^Zr]Zr-labeled anti-MSLN PET-tracers within HCT116 tumors is shown in Figure 5. To compare the anti-MSLN PET tracer’s distribution within the tumor, we defined their distribution uniformity in eq 1. The VH-Fc based tracers (2A10 and 3C9) both demonstrated greater distribution uniformity than observed with the m912 IgG1 antibody tracer. The [^89^Zr]Zr-VH-Fc 2A10 had a distribution uniformity of 46.0 ± 1.40% that was significantly higher than both the [^89^Zr]Zr-VH-Fc 3C9 (42.3 ± 1.54%, *p* = 0.013) and [^89^Zr]Zr-m912 (37.1 ± 3.00%, *p* = 0.002) by an unpaired *t* test. In addition, the [^89^Zr]Zr-VH-Fc 3C9 distribution uniformity was significantly higher (*p* = 0.021) than the antibody-based tracer, [^89^Zr]Zr-m912.

### *Ex Vivo* Biodistribution

**Figure 6:**
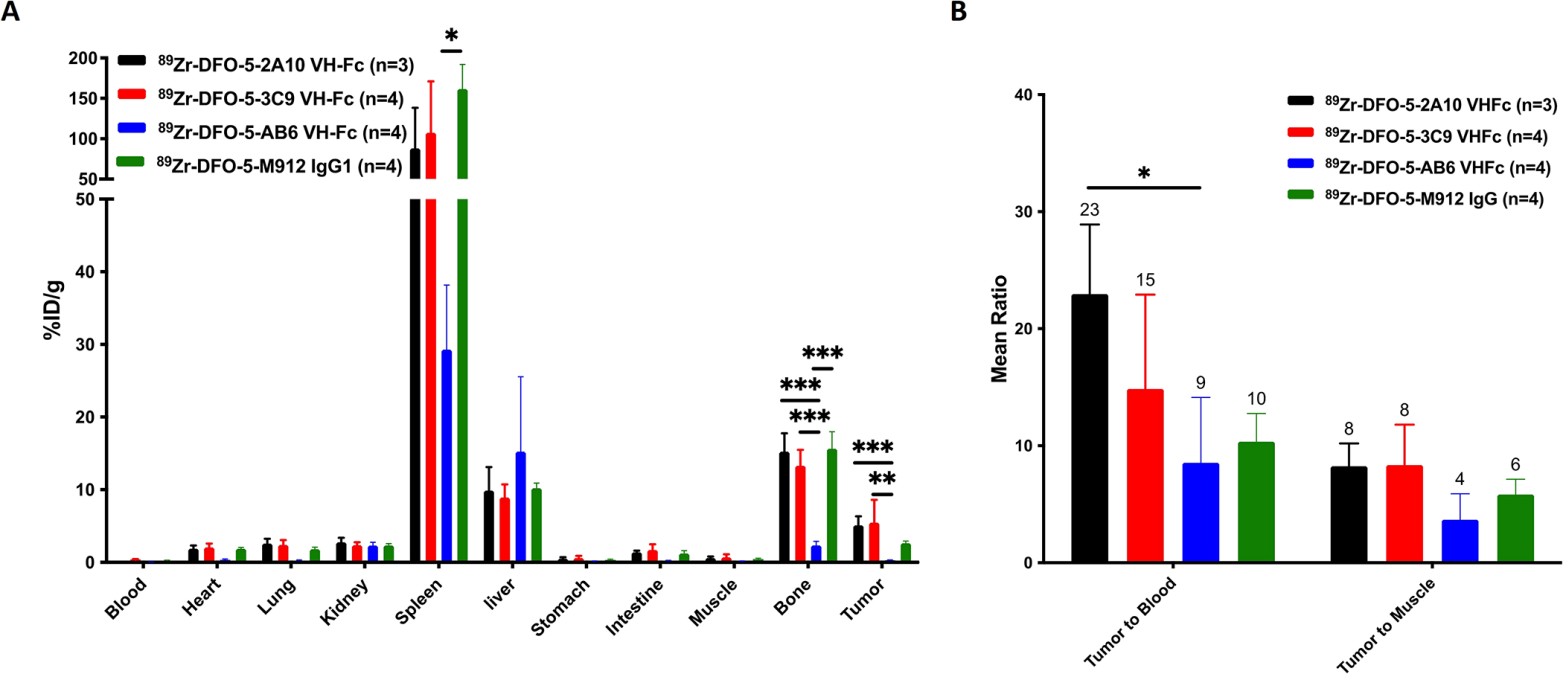
(A) Biodistribution studies at 6-days p.i. of anti-MSLN PET tracers and isotype control. (B) Tumor to blood and tumor to muscle ratios 6-days p.i.

Following the terminal PET-imaging time point (6-days postinjection), *ex vivo* biodistribution studies were performed (Figure 6A). These studies supported the PET findings, with accumulation in the tumor for the anti-MSLN radioconjugates as well as in the liver, spleen, and bone/marrow. Specific accumulation was observed in the tumor for the anti-MSLN agents as compared to the nontargeted control, Ab6 (0.29 ± 0.03%ID/g): m912 (2.64 ± 0.30%ID/g; *p* = 0.598), 3C9 (5.48 ± 3.13%ID/g; *p* = 0.006), and 2A10 (5.10 ± 1.22%ID/g; *p* = 0.017). Accumulation of the anti-MSLN VH-Fcs were higher than the m912 IgG1, but significance was not observed. The tumor-to-blood ratios were highest for the anti-MSLN VH-Fc radioconjugates, with the 2A10 ratio being the highest at 23 followed by 3C9 at 15, m912 at 10, and Ab6 at 9 (Figure 6B); significance was only observed between 2A10 vs Ab6 (*p* = 0.035). The 2A10 and 3C9 radioconjugates demonstrated the highest tumor-to-muscle ratio with a ratio of 8 while the m912 had a ratio of 6 that was only slightly higher than the nontargeted control Ab6 with a ratio of 4, significance was not noted. The highest uptake for all the PET tracers was observed in the spleen: m912 (162 ± 30.2%ID/g; p = 0.005), 3C9 (107 ± 63.5%ID/g), 2A10 (88.0 ± 50.2%ID/g), and Ab6 (29.2 ± 8.88%ID/g) with significant differences observed between m912 and Ab6, the negative control. Similarly, the anti-MSLN PET-tracers demonstrated significantly higher uptake in the femur as compared to the nontargeted Ab6 (2.34 ± 0.57%ID/g): m912 (15.6 ± 2.33%ID/g; *p* ≤ 0.001), 3C9 (13.3 ± 2.19%ID/g; *p* ≤ 0.001), and 2A10 (15.2 ± 2.52%ID/g; *p* ≤ 0.001). Ex vivo biodistribution studies of the 2A10 and m912 tracers in the presence of Fc block resulted in higher uptake in the blood (*p* ≤ 0.001) and tumor (*p* ≤ 0.003) and decreased uptake in the spleen (*p* ≤ 0.005) and bone/marrow (SI Figure 9). Furthermore, 2A10-Fc Block (10.6 ± 0.97%ID/g; *p* ≤ 0.01) had significantly higher uptake in the tumor than m912-Fc Block (7.59 ± 1.53%ID/g).

## Discussion

Mesothelin (MSLN) is highly expressed in a variety of cancers including colorectal cancer, with limited expression in normal tissues,^4,21^ making it a promising target for therapy.^2,22^ Antibody-dependent therapies targeting MSLN, including antibody drug conjugates (ADC) are being evaluated in clinical trials; however, they have had minimal improvements in therapeutic outcomes.^6−10^ The long circulation time and limited tumor penetration of full-length antibodies contribute to the limited therapeutic response. By contrast, domain based anti-MSLN therapeutics, such as domain drug conjugates (DDC), have the potential to improve the therapeutic outcomes of anti-MSLN therapies due to their more appealing pharmacokinetic and penetration properties. Previously, we isolated the high-affinity anti-MSLN VH domain, 3C9, from a phage displayed library. It was modified as a VH-Fc fusion protein and conjugated to monomethyl auristatin E for DDC therapy. The resulting DDC demonstrated promising therapeutic responses in mice bearing MSLN-expressing tumors.^16^ While the initial results were promising, signs of toxicity (weight loss) were observed at higher doses, highlighting the need to develop tools to assess and compare a variety of doses or alternative domain-based targeting agents. The development of anti-MSLN companion PET-agents in parallel to anti-MSLN therapies provides tools to identify tumors that will best respond to anti-MSLN therapy. For example, the anti-MSLN ADC agent, Anetumab ravtansine, was evaluated in a mouse model of uterine cancer, and it was found that only tumors with high MSLN expression demonstrated a compete response.^23^ PET imaging can be used to assess MSLN expression within tumors. In addition, tumor penetration together with gross tumor accumulation better predicted response to ADC in models of metastatic castrate-resistant prostate cancer as the tumor growth, targeted expression levels, and tumor uptake of the ADC’s companion PET agent demonstrated a correlation between expression, uptake, and ADC efficiency.^24^ Preclinical PET-imaging can help evaluate novel domain based anti-MSLN targeting agents by assessing their pharmacokinetics profiles to ensure improved accumulation in the tumor with minimal accumulation in normal tissues as compared to antibody-based agents.

Here, we identified the high affinity and aggregation resistant 2A10 VH domain by library panning with competitor IgG1 m912 (Bayer licensed).^10^ We demonstrated that it likely binds to a different epitope of MSLN compared to our previously isolated anti-MSLN VH domain, 3C9. Furthermore, we demonstrate that the 2A10 VH domain likely binds to an epitope similar to that of the clinical benchmark anti-MSLN antibody IgG1 m912. We opted to initially evaluate the VH domains as VH-Fc fusion proteins due to our previous work investigating the 3C9 VH-Fc for domain-based drug conjugate therapy.^16^ Comparison through PET-imaging of the novel VH-Fc fusion protein, VH-Fc 2A10, to our previously developed anti-MSLN fusion protein, VH-Fc 3C9, and IgG1 m912, allows us to assess the capabilities of PET-imaging to compare the pharmacokinetics of epitope-specific VH domain-based PET-agents as well as compare the difference between a lower molecular weight anti-MSLN PET-tracer (VH-Fc, 80 kDa) to a fully intact antibody (150 kDa).

Herein, the newly developed anti-MSLN 2A10 VH-Fc fusion protein was modified to present DFO for zirconium-89 radiolabeling alongside the previously developed 3C9 VH-Fc fusion protein; m912, an IgG1 antibody, and the negative control, Ab6 VH-Fc. All VH-Fcs and IgG1 m912 were successfully modified and radiolabeled with zircnoium-89 in high yields for PET-imaging. The resulting anti-MSLN PET-tracers ([^89^Zr]Zr-2A10 VH-Fc, 3C9 VH-Fc, and m912) easily distinguished the MSLN-expressing HCT116 tumors as compared to the nontargeted control, [^89^Zr]-Ab6-VH-Fc, which was not able to distinguish the tumor from background (Figure 3 and SI Figure 6). The anti-MSLN ∼ 80 kDa VH-Fcs PET-agents demonstrated improved accumulation (Figure 3 and 6A) within the MSLN-positive HCT116 tumor compared to the 150 kDa IgG1 PET-agent, [^89^Zr]Zr-m912. In addition, microscale analysis (Figure 5) indicated that VH-Fc agents were better able to penetrate the tumor with a higher distribution uniformity than the IgG1 m912 PET-tracer. Furthermore, the [^89^Zr]Zr-2A10 VH-Fc tumor accumulation, ratios (tumor:muscle/heart/blood), and distribution uniformity indicate that the targeted epitope of the 2A10 VH domain may be better suited than the epitope targeted by the 3C9 VH domain. These results are encouraging as it highlights the ability of PET-imaging to assess VH domains as compared to full-length antibodies, as well as potentially allows us to compare VH domains directed to different epitopes of the same target.

The initial evaluation and comparison of the 2A10 and 3C9 domains are presented here as modified VH-Fc fusion proteins for PET-imaging. The performances of both anti-MSLN VH-Fc fusion proteins as PET-tracers are promising, demonstrating improved tumor accumulation and retention compared to an IgG1-based PET agent. Higher accumulation in the liver as compared with the kidneys indicates that clearance occurs via the liver. The PET imaging as well as the *ex vivo* biodistribution of the anti-MSLN agents showed high accumulation in the bone/marrow and spleen. The 2A10-VH-Fc and 3C9-VH-Fc are not cross-reactive to murine MSLN, indicating the bone/marrow and spleen uptake is associated with nonspecific binding. A portion of uptake in the bone, particularly at later time points, is likely associated with free Zirconium-89, which is known to be lost from the DFO chelator and relocate to the bone.^25,26^ The DFO chelator was selected initially as it is the most established chelator for [^89^Zr]Zr-labeled radioimmunoconjugates, and while the *in vivo* stability studies and PET imaging demonstrated that the stability of anti-MSLN PET tracers was adequate for distinguishing MSLN-positive tumors; however, future works will explore alternative chelators or PET radioisotopes to reduce dechelation of Zirconium-89. Furthermore, [^89^Zr]Zr-Ab6 VH-Fc, isotype control, distribution demonstrated significant uptake in the spleen and bone/marrow as compared to background supporting that a portion of the accumulation in the spleen and bone/marrow may be attributed to binding of the Fcs to the FcγRs found in myeloid cells/lymphocytes^27^ and as indicated in the MPA assay (Figure 1C). Fc-specific binding was confirmed by performing Fc blocking studies, which resulted in reductions in spleen and bone/marrow uptake while increasing tumor accumulation of [^89^Zr]Zr-2A10 and [^89^Zr]Zr-m912. In this work, the Fc provided a moiety that helped extend the half-life of the anti-MSLN VH domain-based agents, as the half-lives of the anti-MSLN VH domains (3C9 and 2A10) alone were associated with rapid pharmacokinetics. Future works will aim to explore alternative half-life-extending (HLE) moieties to modify VH domain-based agents to avoid the high FcγR-related accumulation. However, while high non-MSLN binding and loss of zirconium-89 were observed, these data still demonstrates that modified HLE epitope-specific VH domain-based agents can be assessed through PET imaging and are encouraging that the future design and refinement of VH-HLE based agents can be rapidly and noninvasively assessed through PET-imaging.

## Conclusions

The newly isolated antibody VH domain (2A10) with high affinity to MSLN, aggregation resistance, and good specificity was successfully modified as a VH-Fc fusion protein and radiolabeled with zirconium-89. The resulting anti-MSLN VH-Fc PET-tracer was evaluated alongside PET-imaging tracers utilizing a previously developed VH-Fc fusion protein (3C9) and a clinically relevant anti-MSLN IgG1 (m912) as well as a nontargeted VH-Fc fusion protein (Ab6). The resulting anti-MSLN PET-imaging tracers, VH-Fcs and IgG1, demonstrated specific uptake in the MSLN-expressing HCT116 tumors. The *in vivo* performance of the VH-Fc anti-MSLN PET-tracers showed more rapid and greater accumulation and distribution uniformity within the tumor than the full-length IgG1 m912-based PET-imaging agent. Furthermore, the newly developed anti-MSLN VH domain, 2A10,-based PET-imaging tracer showed superior performance compared to the previously developed VH domain, 3C9. These data are encouraging for the continued development and translation of antibody domains for use as targeted PET-imaging agents for MSLN-positive cancers. In addition, the *in vivo* assessment of distributions and tumor penetrations of high affinity anti-MSLN antibody domains through PET-imaging will help guide designs of antibody domain-based chemotherapeutic drug conjugates for treatment of MSLN-expressing cancers.

## Experimental Procedures

### Materials

All chemicals were purchased from Sigma-Aldrich Chemical Co. (St. Louis, MO, USA) or Thermo Fisher Scientific (Pittsburgh, PA, USA), unless otherwise specified. Human mesothelin (MSLN) (296–580) protein (MSN-H522a) was purchased from Biosystems Acro (Newark, DE, USA) or generated in-house (296–606). The [p-SCN-Bn-DFO] chelator was purchased from Macrocyclics, Inc. (Dallas, TX, USA). Zirconium-89 oxalate was purchased from the University of Wisconsin (Madison, WI, USA) or Washington University (St. Louis, MO, USA). The HCT116, a human colorectal cancer cell line, was obtained from the American Type Culture Collection (ATCC, Manassas, VA, USA). Cells were grown in McCoy’s media supplemented with 10% fetal bovine serum and 1% penicillin-streptomycin and incubated in a 5% CO_2_ atmosphere at 37 °C and were routinely tested for mycoplasma.

### Generation and Evaluation of 2A10 VH Domain and VH-Fc Fusion Proteins

The VH domains (2A10, 3C9, Ab6) and their fusion proteins were produced and characterized as previously described^14,15^ (see SI). In brief, the anti-MSLN VH domains were identified from a previously constructed large-scale (1011) human antibody VH domain library based on thermostable antiaggregation scaffolds for phage display. For the conversion of the VH domains to VH domain fusion proteins, the VH gene was reamplified and recloned into pSectaq vector containing human IgG1 Fc fragment. The VH-Fc proteins were expressed in the Expi293 expression system (A14635, Thermo Fisher Scientific, Pittsburgh, PA, USA) and purified by protein A resin (GenScript, Piscataway, NJ, USA). Protein purification and buffer exchange were completed using a PD10 desalting column (GE Healthcare, Chicago, IL, USA). Protein purity was estimated as >95% by SDS-PAGE. To characterize the VH domain and fusion proteins, ELISA assays, size exclusion chromatography (SEC), surface plasmon resonance (SPR), and membrane proteome array (MPA) were conducted as previously described.^16,19^

### Conjugation, Radiolabeling, and *In Vivo* Stability of Anti-MSLN Antibody and VH-Fc Fusion Protein

The anti-MSLN antibody (m912) and VH-Fcs (2A10 and 3C9) as well as the untargeted VH-Fc (Ab6) were conjugated p-SCN-Bn-DFO at a 1:5 molar ratio as previously describe^28^ (see SI). Chelator to protein ratios were determined as previously described.^29^ Radiolabeling, as described in the SI, was performed using [^89^Zr]Zr-oxalate. Radiolabeling yield (RLY) and purity (RLP) were determined by iTLC-SG; 10 mM EDTA. All radiolabeled conjugates were buffer exchanged with PBS using a centrifuge filtering cartridge (Vivaspin 6, 30 kDa MWCO) prior to *in vivo* injections. *In vivo* stability studies for the [^89^Zr]Zr-2A10, −3C9, and -m912 were performed in mice at 24 and 48 h (see SI).

### MSLN Expression in HCT116 Cells and Tumor Model

Expression of MSLN in the HCT116 cells and tumors as well as the spleen, kidney, liver, marrow, and bone were evaluated (see SI). Expression in the HCT116 cells were characterized by western blotting.^4^ To assess expression (heterogeneous vs homogeneous) of MSLN within HCT116 tumors, we evaluated fixed tumor slices by immunohistochemistry. Select murine tissues were evaluated by IHC for MSLN-expression.

### PET Imaging

PET-imaging studies were performed in NCG mice (8–10 weeks) bearing HCT116-tumors using an Inveon small animal microPET/CT (Siemens Molecular Imaging, Knoxville, TN, USA) as previously described.^30^ The mice were injected intravenously (i.v.) with the radioconjugates (see Table 1) and imaged at 90 min (VH-Fc radioconjugates only); 18, 48, 96, and 144 h (see SI for imaging parameters). Additional PET-imaging studies were performed on the 2A10 VH-Fc and m912 radioimmunoconjugates in the presence of 25x excess of an irrelevant antibody to serve as a Fc Block at 90 min; 24, 48, and 120 h (see SI Table 1). Volumes of interest (VOIs) were defined by CT for the following organs: tumor, heart, vena cava (blood), and muscle. The uptake of the tracer in normal tissues and tumor are presented as SUV_mean_.

### iQID Imaging

The iQID-camera system^31^ was used to image and quantify the activity concentration and distribution of the [^89^Zr]Zr-labeled 2A10 VH-Fc, 3C9 VH-Fc, and m912 (see SI). Briefly, sectioned tissue samples were placed on a scintillator sheet BioMaxTranScreen HE (Carestream Health Inc., Rochester, NY, USA) and imaged in an iQID-camera system. The images were processed and analyzed using the MATLAB R2023a software (MathWorks Inc., Natick, MA, USA) and ImageJ2 version 2.9.0/1.53t (National Institutes of Health, Bethesda, MD, USA). The distribution uniformity within a tumor section is defined as the percentage of the area that has an activity that is higher than or equal to the average activity of the whole section.
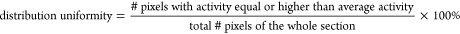
1

### *Ex Vivo* Biodistribution Studies

Biodistribution studies were conducted as previously described following PET imaging.^30^ Select tissues (see SI) were harvested, weighed, and measured. The percentage of injected dose per gram (%ID/g) was calculated using the injected activity converted to CPMs (activity (DPMs) × efficiency for individual radioisotopes) and decay corrected.

### Statistical Analysis

All data are presented as mean ± SD. Biodistribution groups were compared using one-way analysis of variance(ANOVA) followed by Tukey’s HSD for pairwise comparisons if the ANOVA showed detectable difference. *P* values were adjusted for multiple testing. SUV means were assessed by a mixed model with a random intercept to address repeated measures and pairwise tests for marginal group means at each time point. The SUV mean background value was set at 0.1, so if a value <0.1, it was changed to 0.1. Distribution uniformities were compared using a two-tail *t* test. Type I error rate was set at 0.05. Statistical analysis was performed by Ziyu Huang using R version 4.3.1.

## Supporting Information Available

Detailed materials and methods; additional figures for SPR, SEC profiles, western blots, IHC and Fc blocking studies; and tables for Fc blocking studies and *in vivo* stability (PDF)
