# Supplementary material for: Assessment of Novel Mesothelin-Specific Human Antibody Domain VH-Fc Fusion Proteins-Based PET Agents
Source: ACS Omega. 2023 Nov 8;8(46):43586–95. doi: 10.1021/acsomega.3c04492 (PMC10666227; doi:10.1021/acsomega.3c04492)
Supplement: Supplementary file 1 — ao3c04492_si_001.pdf [file ao3c04492_si_001.pdf]

SUPPORTING INFORMATION

Assessment of novel mesothelin specific human antibody domain VH-Fc fusion proteins-based PET agents

Zehua Sun[†*], Ambika P. Jaswal[‡*], Xiaojie Chu[†*], Harikrishnan Rajkumar[§], Angel G. Cortez[§], Robert Edinger[§], Max Rose[§], Anders Josefsson[§,#], Abhinav Bhise[§,#], Ziyu Huang[§], Rieko Ishima[¶], John W Mellors[†], Dimiter S. Dimitrov[†**], Wei Li [†**], and Jessie R. Nedrow[§, #**]

[†]Center for Antibody Therapeutics, Division of Infectious Diseases, Department of Medicine, University of Pittsburgh School of Medicine, Pittsburgh, PA, USA

[‡]Department of Neurological Surgery, University of Pittsburgh School of Medicine, Pittsburgh, PA, USA

[§]Hillman Cancer Center, University of Pittsburgh School of Medicine, Pittsburgh, PA, USA

[¶]Department of Structural Biology, University of Pittsburgh School of Medicine, Pittsburgh, PA, USA
[#]Department of Radiology, University of Pittsburgh School of Medicine, Pittsburgh, PA, USA

[*]Authors contributed equally to manuscript

[**]Co-Corresponding authors

Corresponding Authors:

Jessie R. Nedrow, Ph.D.
5117 Centre Avenue, Suite G. 17b, Pittsburgh, PA 15213, USA
Phone Office: +1 (412) 623-7239; Email: nedrowj@upmc.edu

Wei Li, Ph.D.
S843 Scaife Hall, 3550 Terrace Street, Pittsburgh, PA 15261, USA
Phone Office: +1 (412) 383-4703; Email: liwei171@pitt.edu

Dimiter S. Dimitrov, Ph.D.
S843 Scaife Hall, 3550 Terrace Street, Pittsburgh, PA 15261, USA
Phone Office: +1 (412) 383-4702; Email: mit666666@pitt.edu

SUPPORTING INFORMATION MATERIALS AND METHODS

Generation and Evaluation of 2A10 VH domain and VH-Fc fusion proteins.

Identification of VH domains that compete with IgG1 m912. The anti-MSLN VH domains were identified from a previously constructed large-scale (10^{11}) human antibody VH domain library based on thermostable anti-aggregation scaffolds for phage display [1, 2]. Panels of binders were panned and isolated from this antibody VH domain library against MSLN. Three rounds of panning were performed, and after each round of panning, phages were eluted with increasing concentration of IgG1 m912 (10, 50, and 100 nmol, respectively). Phage based flow cytometry was performed for binder selection. IgG1 m912 was purified by Expi 293 expressing system (A14635, Thermo Fisher Scientific)[7].

Production of isolated MSLN and VH domains, and fusion proteins. Human MSLN, residues from 296-606 (https://www.uniprot.org/uniprot/Q13421), and 2A10 VH domain were generated as described previously for the MSLN specific VH domain, 3C9[2]. The coding DNA, in the pComb3x vector, was expressed in Escherichia coli HB2151 at 30 °C, with 1 mmol IPTG induction, for 16 h. The non-specific Ab6 VH domain was produced from HB2151 transformed p3x-VHAb6 plasmid. Harvested cells were lysed with Polymyxin B (Sigma-Aldrich, St. Louis, MO, USA). Proteins were purified from the supernatant with Ni-NTA column (GE Healthcare, Chicago, IL, USA). For the conversion of the VH domains to VH domain fusion proteins, the VH gene was re-amplified and re-cloned into pSectaq vector containing human IgG1 Fc fragment. The VH-Fc proteins were expressed in the Expi293 expression system (A14635, Thermo Fisher Scientific, Pittsburgh, PA, USA) and purified by protein A resin (GenScript, Piscataway, NJ, USA). Protein purification and buffer exchange was completed using a PD10 desalting column (GE Healthcare, Chicago, IL, USA). Protein purity was estimated as >95% by SDS-PAGE.

ELISA Assays. The assay was conducted as previously described[16]. In brief, the mesothelin coated plates were prepared, and after blocking, the serially diluted biotinylated VH-Fcs or IgG1 were added into wells for incubation (Biotinylating Kit - Abcam, CAT# ab201795). For the biotin-labeled protein binding kinetics, the protein was titrated from 1000 nmol to 0.06 nmol in 1:4 serial dilutions. Next, 50 μl of the diluted antibody was added to duplicate wells. In the ELISA with biotin-labeled protein, HRP-Conjugated Streptavidin (ThermoFisher Scientific, CAT# N100) was used at a dilution of 1:1000. In the competition ELISA, a designated concentration of competing IgG1 or VH-Fc protein was added to wells. After a 1 h incubation at 37 °C, the wells were washed four times with PBST (PBS+0.05% Tween 20). In the cross-reactive ELISA, the mouse MSLN (Sino Biological, 5A0830-M08H) were coated into plates overnight, and after blocking, then serially diluted VH-Fcs or IgG1 were added and incubated at 37 °C for 1h. After washing with PBST, the bound IgG1 or VH-Fc protein was detected using anti-human Fc-HRP mAb (1:1000) (Sigma) for 1 h at 37 °C. Following another wash with PBST, substrate ABTS (50 μl/well) was added, and the reaction was read at 405 nm.

Size Exclusion Chromatography (SEC). Estimation of apparent molecular sizes of the VH and the VH-Fc 2A10 was performed by injecting 150 μL filtered proteins (1-2 mg/mL) to the Superdex 200 Increase 10/300 GL chromatography column (GE Healthcare, Cat. No. 28990944) equilibrated with PBS buffer at a 0.4 mL/min flow rate. Gel filtration calibration kit (Cytiva, # 28403842), containing protein molecular mass standards, was used to determine the molecular size of the injected proteins.

Surface Plasmon Resonance (SPR). The kinetics of the antibody fragments were determined using SPR as previously described[13, 21]. Briefly, human MSLN (Advance BioMatrix 5123-0.1 mg) or murine MSLN (Sino Biological, 5A0830-M08H) was immobilized onto a CM5 sensor chip (GE Healthcare, BR100012) by amine coupling. The VH domain and domain-based agents as well as the IgG1 were diluted in HBS-EP buffer (10 mmol HEPES, 150 mmol NaCl, 3 mmol EDTA, and 0.005% surfactant P20, pH 7.4) and evaluated by SPR at increasing concentrations (Supplemental Figure 1 and 2). After each sample injection, the surface was regenerated by injection of regeneration solution (10 mmol Glycine/HCl buffer, with 10% Glycerol at pH 2.0). The kinetic values, k_a, k_d, and K_D were calculated using the BiacoreX100 Evaluation Software (GE Healthcare).

Membrane Proteome Array (MPA) was performed by Integral Molecular, Inc. (Philadelphia, PA, USA) to specificity test VH-Fc 2A10 using the MPA platform, using the previously described sample preparation protocol[16]. The MPA comprises 6,000 different human membrane associated protein clones. Before specificity testing, optimal antibody concentrations for screening were determined and cell specific binding was confirmed. Binding across the protein library was measured on an iQue3 (Ann Arbor, MI). To ensure data validity, each array plate contained positive (Fc-binding; MSLN protein) and negative (empty vector) controls. Identified targets were confirmed in a secondary flow cytometric experiment with serial diluted antibody of test. The identity of each target was confirmed by sequencing.

Conjugation and radiolabeling of anti-MSLN antibody and VH-Fc fusion protein.

Conjugation of anti-MSLN antibody and VH-Fc fusion protein. The anti-MSLN antibody (m912) and VH-Fcs (2A10 and 3C9) as well as the untargeted VH-Fc (Ab6) were conjugated p-SCN-Bn-DFO using methods previously described with minor modifications. The p-SCN-Bn-DFO was used at a 1:5 molar ratio[3]. The conjugation buffer for the p-SCN-Bn-DFO conjugations was 500 mmol $NaHCO_3$, 20 mmol Na_2CO_3, NaCl 1.5 mol, and 10 mmol EDTA. The chelators, antibody or VH-Fcs, and buffer were combined, incubated at 37 °C for 1 h then purified by SEC centrifugation with sterile PBS. Chelator to protein ratios were determined as previously described[4].

Radiolabeling of of [^{89}Zr]Zr-labeled anti-MSLN Conjugates. A stock solution of [^{89}Zr]Zr-oxalate was prepared at 37 MBq(1 mCi)/50 µL 1 mol oxalic acid based on activity at time of production. An aliquot of the stock solution (13.5-13.9 MBq, 25 µL) was added to an acid washed Eppendorf tube containing 175 µL of 1 mol HEPES buffer (pH=8) and 100 µg (57-116 uL) of anti-MSLN antibody or modified domains. The mixture was incubated for 60 min at 37 °C. Radiolabeling yield (RLY) and purity (RLP) were determined by iTLC-SG; 10 mM EDTA. All radiolabeled conjugates were buffer exchanged with PBS using a centrifuge filtering cartridge (Vivaspin 6, 30 kDa MWCO) prior to *in vivo* injections.

***In vivo* stability of VH-Fc domains.** Purified radioimmunoconjugates (2 – 3 MBq in 100 µL of PBS, pH 7.4) were intravenously administered to healthy CD1 IGS mice (12-14 weeks, 24 – 32 g, n = 2). Blood samples (75 – 100 µL) were withdrawn from the cheeks at 24 h and 48 h post-injection (p.i.). Subsequently, the collected blood was loaded onto preactivated PD10 columns (Cytiva, Sephadex G-25M) followed by elution with PBS, resulting in the collection of 25 fractions. The radioactivity present in each fraction (0.5 mL/tube for fractions 1 – 20, and 1 mL/tube for fractions 21 – 25 fractions) was measured utilizing a WIZARD^2 gamma counter (PerkinElmer, Waltham, MA, USA). The percentage of intact radioimmunoconjugates was determined by calculating the ratio of counts from the intact peak to the total counts of the chromatograms, and this ratio was then multiplied by 100%. The PD10 columns were activated through a 1% BSA (5 mL) followed by PBS (25 mL).

MSLN expression in HCT116 cells and tumor model.

Western Blot. The western blot analysis was performed as previously described with modifications[5]. Briefly, the HCT116 cell lysate (30 µg) was loaded on the gel and ran on were loaded on denaturing 4-20% TGX precast gels (BioRad Laboratories, Hercules, CA) and run using a BioRad Mini electrophoresis system. Proteins were transferred to a mini-nitrocellulose membrane. Membranes were blocked in 5% non-fat dry milk in Tris-buffered saline 0.1% overnight at 4 °C. Membranes were incubated overnight at 4 °C with 2.5 µg/mL of the primary antibodies [anti-mesothelin rabbit monoclonal antibody SAB55001430 (Sigma, St. Lois, MO, USA)] in 5% non-fat dry milk TBS and then incubated in a secondary antibody-horseradish peroxidase conjugate (BioRad) for 1 h at 25 °C. The membranes were developed using ECL western blotting detection reagents (EMD Millipore, Billerica, MA), detected using BioRad ChemiDoc MP gel imager and quantified using BioRad Image Lab software.

Immunohistochemistry (IHC) was performed for the HCT116 tumors to assess distribution of MSLN within the tumor as well as the following normal tissue: spleen, kidney, liver, marrow, and bone. Tumors were extracted from tumor burden mice and were fixed in 10% formalin. The tumor samples were transferred to 70% ethanol post 48 h. The sections were obtained using a microtome and were transferred to slides. The slides were deparaffinized and rehydrated using a standard histology protocol. Antigen retrieval was performed using a Citrate buffer (Cell Signaling, Danvers, MA, USA). The antibody used was mouse anti-Human,Mouse,Rat – Clone: MSLN/2131 from Fisher Scientific (NMP2724), which was applied using a 1:1000 dilution at room temperature. An IgG2b Kappa Isotype control from Fisher Scientific (14-4732-82) was used at a 1:100 dilution at room temperature, providing a control for non-specific staining. The secondary antibody used was Boost Rat HRP Polymer from (Pacheco, CA, USA). The substrate used was 3,3, Diaminobenzidine + (Cell Signaling, Danvers, MA, USA).

PET-imaging. PET-imaging studies were performed in NCG mice (8-10 weeks) mice bearing HCT116-tumors using an Inveon small animal microPET/CT (Siemens Molecular Imaging, Knoxville, TN, USA) as previously described[6]. The mice were injected intravenously (i.v.) with the radioconjugates (see Table 1, SI Table 1) and imaged with the following parameters: 10 min PET acquisition time, OSEM2D (standard uptake value ((SUV) calculations) and OSEM3D (PET/CT) reconstruction algorithms, voxel size 0.8 mm^3; CT-based attenuation correction, CT exposure settings: 80 kV, 500 µA, 145 ms exposure time, 220° rotation with 120 steps, low magnification, bin 4x4, voxel size 0.8 mm^3; CT reconstruction: Feldkamp algorithm, Shepp-logan reconstruction filter, downsample factor 2, image voxel size: x = 196.43 µm, y = 196.43 µm, z = 196.43 µm. For the Fc block, excess unlabeled Fc block (0.5 mg, irrelevant anti-SARS-CoV-2 IgG1 ab1) was co-injected with the 2A10 VH-Fc and m912 PET agents. PET/CT-images were exported as DICOM to Vivoquant Version (Invicro, Needham, MA, USA). Volume of interests (VOIs) were defined by CT for the following organs: tumor, heart, vena cava (blood), and muscle. The uptake of the tracer in normal tissues and tumor are presented as SUV_{mean}.

iQID-imaging. The iQID-camera system[7] was used to image and quantify the activity concentration and distribution of the $[^{89}Zr]$Zr-labeled 2A10 VH-Fc, 3C9 VH-Fc, and m912. Briefly, the HCT116-tumor bearing mice were sacrificed 18 h post-injection and the MSLN-positive tumors were immediately harvested, embedded in optimal cutting temperature (OCT) and flash-frozen on dry ice (-78.5 °C). The frozen tumors were sectioned using a cryostat TN50 (Tanner Scientific, Sarasota, FL, USA) in 16 µm (tumors) thick sections. The sectioned tissue samples were placed on a scintillator sheet BioMax TranScreen HE (Carestream Health Inc., Rochester, NY, USA), with a total exposure time of 15 h in the iQID-camera system. The images were processed and analyzed using the MATLAB R2023a software (MathWorks Inc., Natick, MA, USA) and ImageJ2 version 2.9.0/1.53t (National Institutes of Health, Bethesda, MD, USA). The distribution uniformity within a tumor section is defined as the percentage of the area, which has an activity that is higher or equal to the average activity of the whole section.

$$Distribution\ Uniformity = \frac{\#Pixels\ with\ activity\ equal\ or\ higher\ than\ average\ activity}{Total\ \#\ pixels\ of\ the\ whole\ section} \cdot 100\% \qquad \text{(Eq 1.)}$$

***Ex vivo* Biodistribution studies.** Biodistribution studies were conducted as previously described in healthy NCG female mice (n=3-4/group) bearing HCT116 subcutaneous tumors [6]. Following PET-imaging, mice were sacrificed at ~6 days and the following organs collected: blood, heart, lungs, kidneys, spleen, liver, stomach (w/ content), intestines (w/ content), muscle, bone (femur w/ marrow) and tumors were harvested, weighed, and measured in an automatic γ-well counter (PerkinElmer 2480 WIZARD2 Automatic Gamma Counter, MA, USA). The percentage of injected activity per gram (%IA/g) was calculated using the injected activity converted to CPMs (Activity (DPMs) x efficiency for individual radioisotopes) and decay corrected.

SUPPORTING INFORMATION FIGURES AND LEGENDS

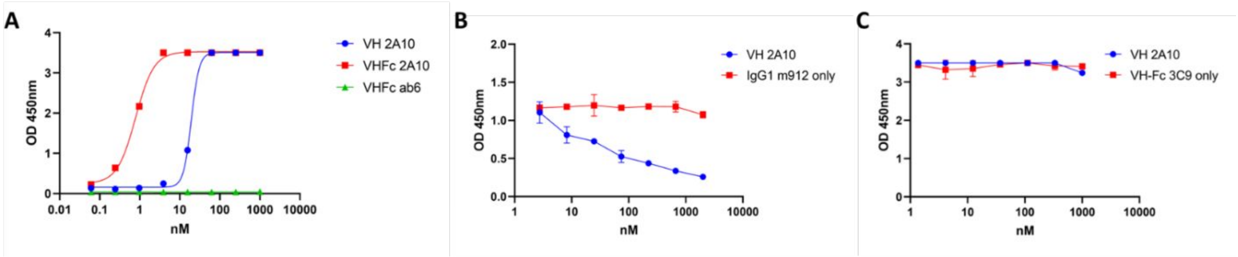

Supporting Information Figure 1. (A) ELISAs of 2A10 VH and VH-Fcs biding to human MSLN as compared to untargeted control, Ab6 VH-Fc. The 2A10 VHh was assessed further by ELISA in competition with (B) IgG1 m912 and (C) 3C9 VH-Fc.

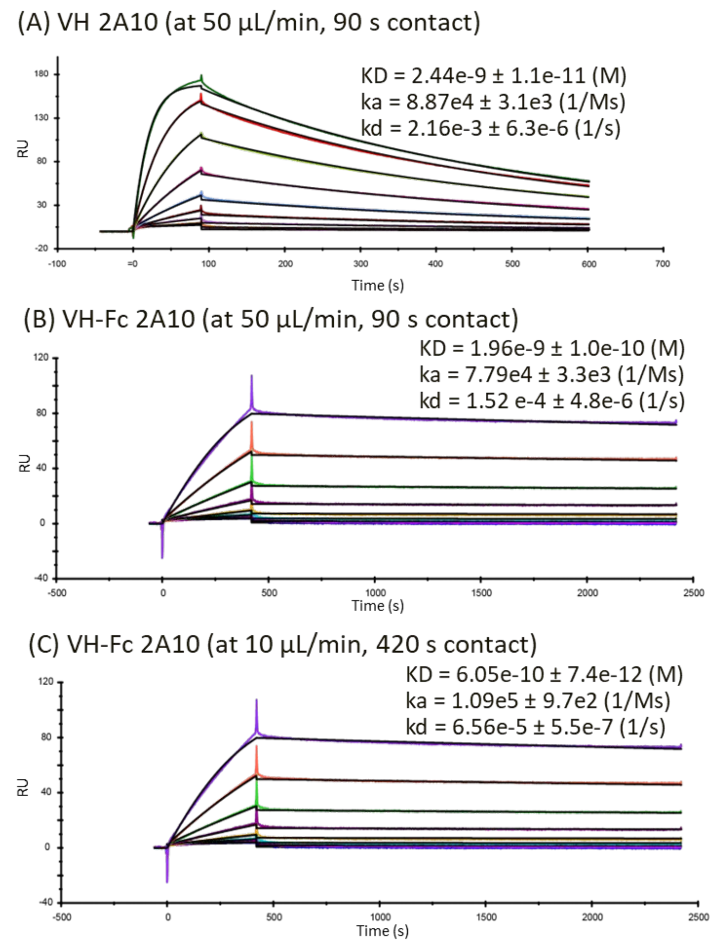

Supporting Information Figure 2. Surface plasmon resonance (SPR) sensograms for evaluating the binding affinity of (A) VH 2A10 and (B, C) VH-Fc 2A10, to MSLN. All the SPR experiments were performed using Biacore X100 instrument (GE Healthcare). The full-length human MSLN in 0.3 µmol solution was injected to be immobilized onto a CM5 sensor chip (Cytiva, BR100012) by amine coupling, at 5 µL/min until to reach 800 response unit. VH 2A10 and VH-Fc 2A10 at 100 nmol and 50 nmol, respectively, were serially diluted in HBS-EP buffer (10 mmol HEPES, 150 mmol NaCl, 3 mmol EDTA, and 0.005% surfactant P20, pH=7.4). In panels (A) and (B), these analytes were injected for 90 sec at a rate of 50 µL/min, followed by dissociation for 600 sec, and regenerated by an injection of 10 mmol Glycine/HCl buffer, with 10% Glycerol at pH=2.0. In panel (C), the analyte was injected at 10 µL/min for 420 sec, and regenerated by an injection of 0.5 mol Gdn HCl, at pH=3.0, after 2000 sec waiting time. The condition (B) was used to compare the binding of VH-Fc 2A10 with VH 2A10 in (A), while the condition (C) was more suitable to investigate VH-Fc forms. Note that the dissociation constants of VH 2A10 and VH-Fc 2A10 were lower than those of VH 3C9 that was previously studied, 2.6 and 7.4 nmol, respectivel[2].

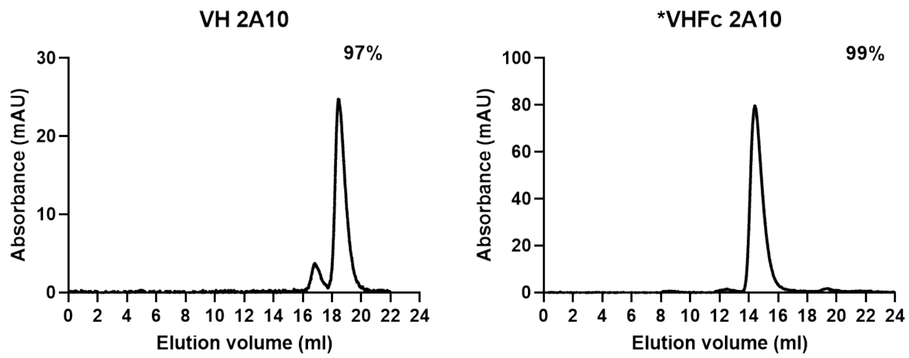

Supporting Information Figure 3. SEC of antibody VH domain 2A10 and the VH-Fc 2A10 fusion protein.

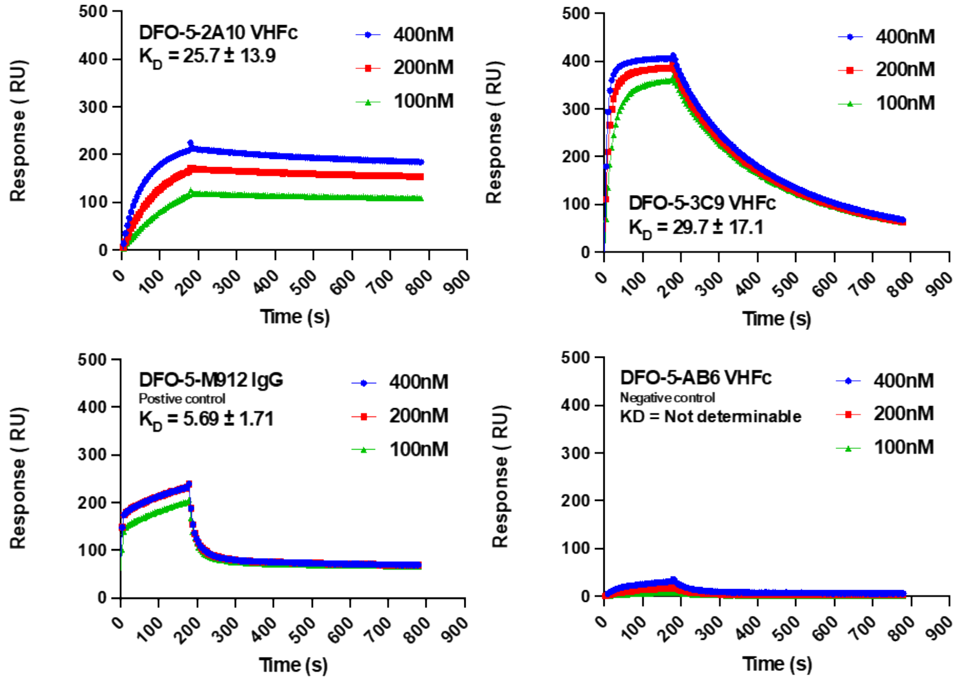

Supporting Information Figure 4. Representative sensograms of concentration binding of DFO conjugated anti-MSLN VH-Fcs and controls to human mesothelin. All the SPR experiments were performed using Biacore X100 instrument (GE Healthcare). Human MSLN (Advance BioMatrix 5123-0.1 mg) in 0.25 μmol solution was injected to be immobilized onto a CM5 sensor chip (Cytiva, BR100012) by amine coupling, at 5 μL/min until to reach 400 response unit. DFO conjugated VH-Fc 2A10, VH-Fc 3C9, VH-Fc Ab6, and IgG1 m912 at 400 nmol were serially diluted in HBS-EP buffer (10 mmol HEPES, 150 mmol NaCl, 3 mmol EDTA, and 0.005% surfactant P20, pH=-7.4) and analytes were injected for 180 sec at a rate of 20 μL/min, followed by dissociation for 600 sec, and regenerated by an injection of 10 mmol Glycine/HCl buffer, with 10% Glycerol at pH=2.0.

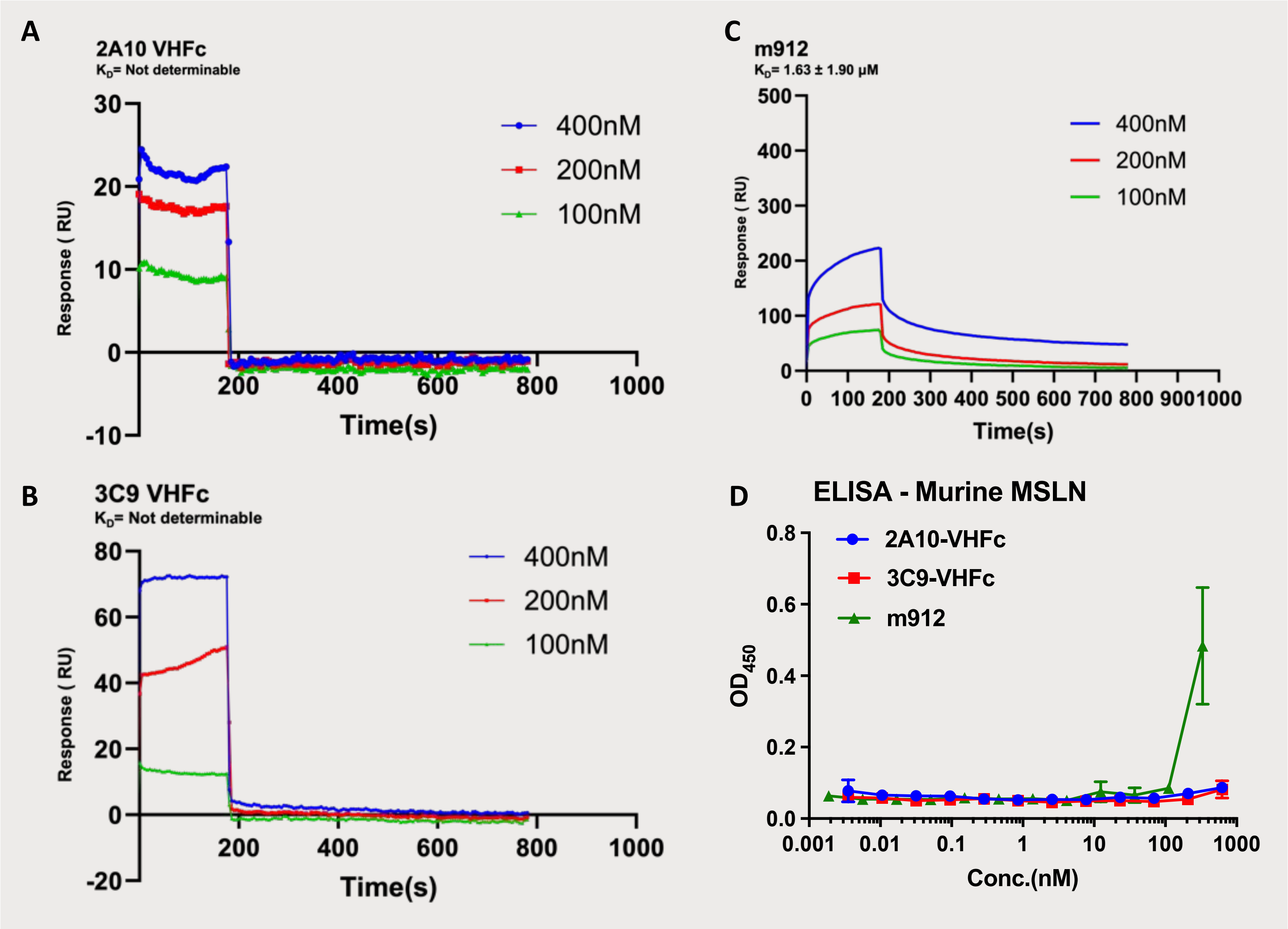

Supporting Information Figure 5. Representative sensograms (A-C) and ELISA (D) of anti-MSLN VH-Fcs and m912 (positive control) to murine mesothelin (Sino Biological, 5A0830-M08H). All the SPR experiments were performed using Biacore X100 instrument (GE Healthcare).

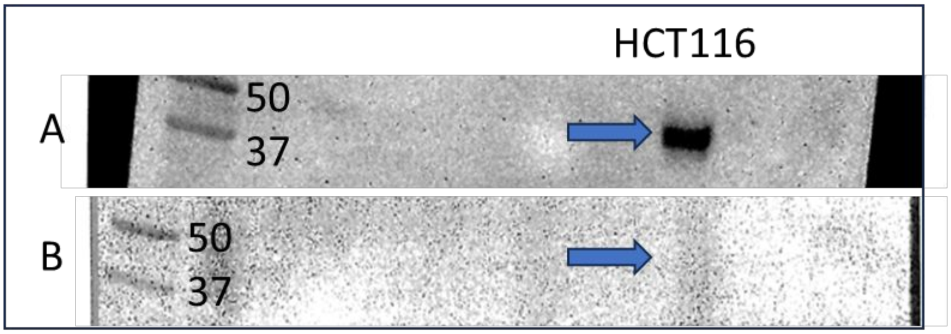

Supporting Information Figure 6. Western blot analysis of MSLN (40 kDa) in human colorectal cancer HCT116 cells. (A) Primary and secondary antibodies (B) Control – No primary antibody

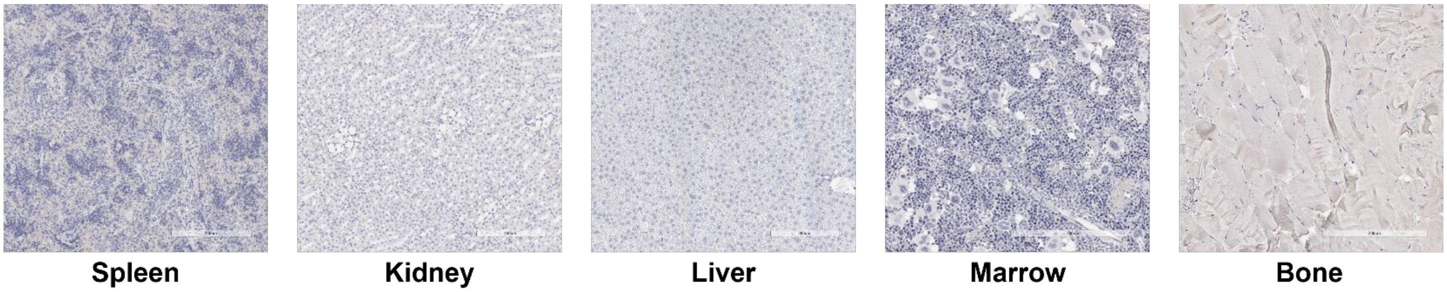

Supporting Information Figure 7. Representative immunohistochemistry of mesothelin (MSLN) expression in select tissue sections. MSLN staining was not observed. Specificity of anti-MSLN was confirmed by an isotype control primary antibody in HCT116 tumors (see Figure 2).

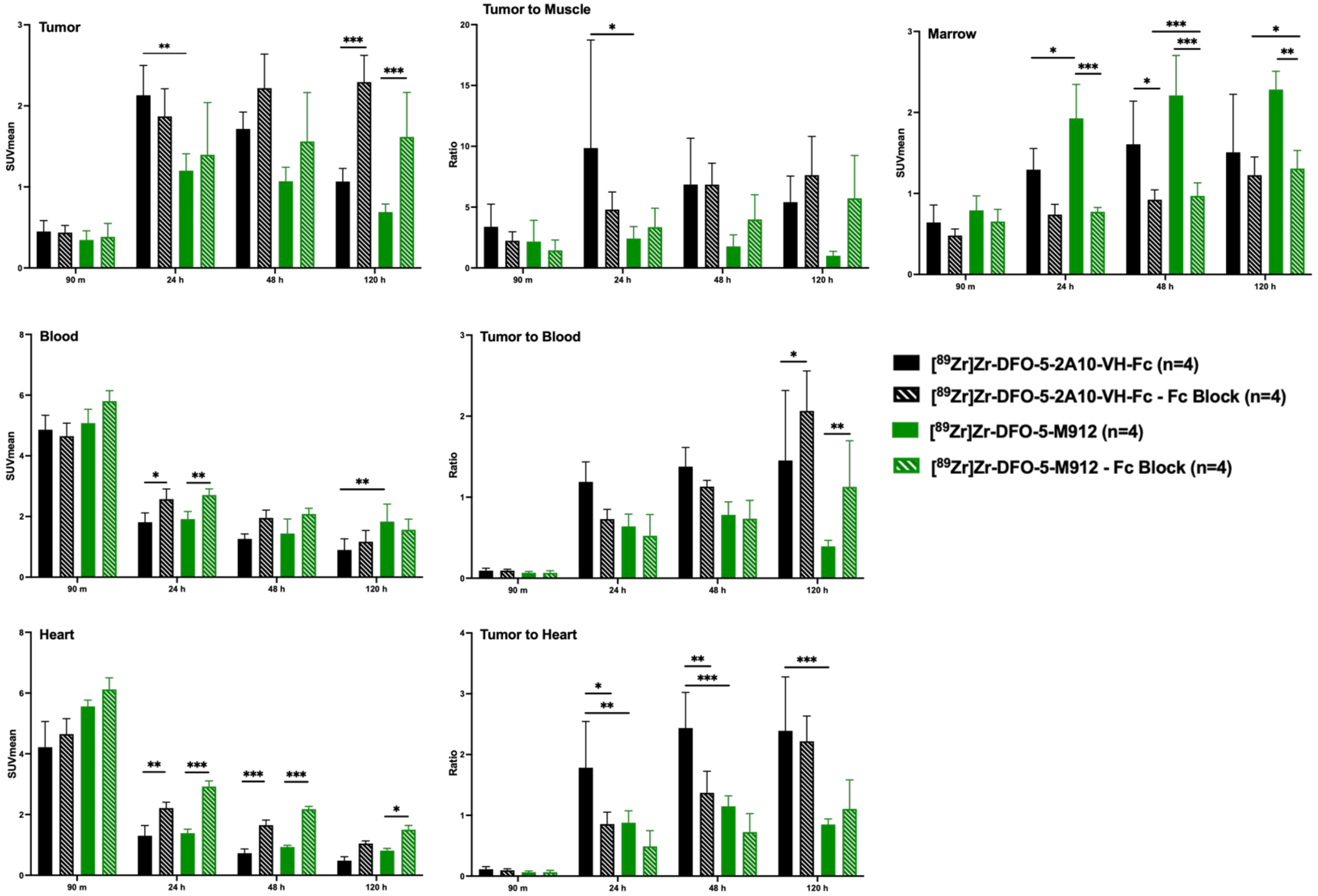

Supporting Information Figure 8. SUV_{mean} of zirconium-89 labeled anti-MSLN PET tracers over a 5-day window with and without Fc Block.

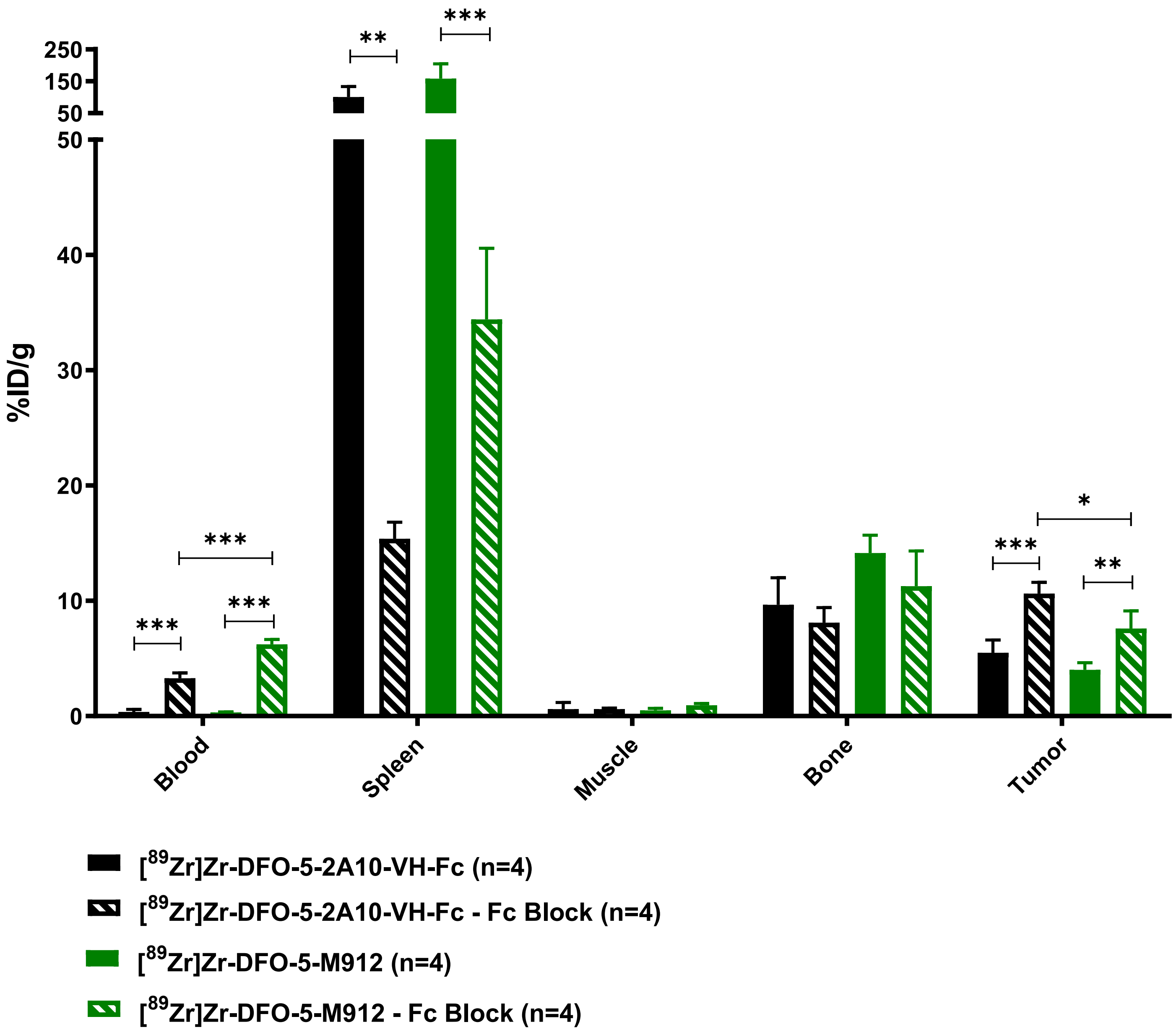

Supporting Information Figure 9. Biodistribution studies at 5-days p.i. anti-MSLN PET tracers with and without Fc Block.

SUPPORTING INFORMATION TABLES

SI Table 1.

SI Table 1. Summary of [^{89}Zr]Zr-labeled radiotracers injected into HCT116-tumor bearing mice for Fc blocking studies

[^{89}Zr]Zr-labeled PET tracer	Activity Injected		Protein Amount (µg/100 µL)	Molar Activity (MBq/µmol)	Number of Mice Injected
	MBq	µCi			
**2A10-VH-Fc**	1.36 – 1.81	37 - 49	20	1.50 ± 0.22	4
**2A10-VH-Fc + Fc Block**	1.77 – 1.89	48 – 51	20	1.37 ± 0.04	4
**M912-IgG1**	1.25 - 1.36	34 – 37	20	2.06 ± 0.04	4
**M912-IgG1 + Fc Block**	1.25 – 1.36	34 – 36	20	1.87 ± 0.08	4

SI Table 2. *In vivo* blood stability

Time Point	24h				48h			
Conjugate	M1	M2	Avg	STD	M1	M2	Avg	STD
[^{89}Zr]Zr-2A10	90.4	92.3	91.3	1.36	84.8	83.8	84.3	0.71
[^{89}Zr]Zr-3C9	92.6	86.4	89.5	4.42	83.4	80.7	82.1	1.90
[^{89}Zr]Zr-M912	84.3	86.1	85.2	1.27	87.3	84.3	85.8	2.15

SI Table 3. SUV_{mean} values for anti-MSLN PET tracers with and without Fc Block

Tracer		Tumor	Heart	Blood	Muscle	Tumor: Muscle	Tumor: Heart	Tumor: Blood
[^{89}Zr]Zr-DFO-5-2A10-VH-Fc (n=4)	90 m	0.45 ± 0.13	4.22 ± 0.85	4.86 ± 0.48	0.16 ± 0.08	3.38 ± 1.86	0.11 ± 0.04	0.09 ± 0.03
	24 h	2.13 ± 0.37	1.30 ± 0.34	1.81 ± 0.31	0.34 ± 0.20	9.85 ± 8.88	1.78 ± 0.76	1.19 ± 0.25
	48 h	1.72 ± 0.21	0.73 ± 0.14	1.26 ± 0.16	0.31 ± 0.15	3.25 ± 3.80	2.44 ± 0.59	1.38 ± 0.24
	120 h	1.07 ± 0.16	0.48 ± 0.13	0.90 ± 0.37	0.23 ± 0.10	5.41 ± 2.14	2.39 ± 0.89	1.45 ± 0.86
[^{89}Zr]Zr-DFO-5-2A10-VH-Fc (n=4) plus Fc Block		Tumor	Heart	Blood	Muscle	Tumor: Muscle	Tumor: Heart	Tumor: Blood
	90 m	0.43 ± 0.09	4.64 ± 0.51	4.65 ± 0.43	0.20 ± 0.05	2.24 ± 0.73	0.09 ± 0.06	0.09 ± 0.02
	24 h	1.87 ± 0.34	2.21 ± 0.19	2.57 ± 0.34	0.41 ± 0.09	4.80 ± 1.45	0.86 ± 0.20	0.73 ± 0.12
	48 h	2.22 ± 0.42	1.65 ± 0.17	1.95 ± 0.26	0.33 ± 0.06	6.85 ± 1.76	1.37 ± 0.35	1.13 ± 0.08
	120 h	2.29 ± 0.33	1.04 ± 0.09	1.17 ± 0.37	0.35 ± 0.18	7.63 ± 3.18	2.22 ± 0.42	2.06 ± 0.49
[^{89}Zr]Zr-DFO-5-m912 IgG1(n=4)		Tumor	Heart	Blood	Muscle	Tumor: Muscle	Tumor: Heart	Tumor: Blood
	90 m	0.35 ± 0.11	5.56 ± 0.21	5.08 ± 0.46	0.21 ± 0.09	2.17 ± 1.74	0.06 ± 0.02	0.07 ± 0.02
	24 h	1.20 ± 0.21	1.39 ± 0.13	1.91 ± 0.25	0.55 ± 0.19	2.41 ± 0.98	0.88 ± 0.20	0.64 ± 0.15
	48 h	1.07 ± 0.17	0.93 ± 0.06	1.44 ± 0.48	0.72 ± 0.33	1.77 ± 0.94	1.15 ± 0.17	0.78 ± 0.16
	120 h	0.69 ± 0.10	0.81 ± 0.07	1.83 ± 0.58	0.77 ± 0.32	1.00 ± 0.36	0.85 ± 0.09	0.39 ± 0.07
[^{89}Zr]Zr-DFO-5-m912 IgG1(n=4) plus Fc Block		Tumor	Heart	Blood	Muscle	Tumor: Muscle	Tumor: Heart	Tumor: Blood
	90 m	0.38 ± 0.17	6.12 ± 0.38	5.80 ± 0.35	0.31 ± 0.15	1.44 ± 0.86	0.06 ± 0.03	0.07 ± 0.03
	24 h	1.40 ± 0.64	2.92 ± 0.19	2.71 ± 0.20	0.43 ± 0.08	3.35 ± 1.55	0.49 ± 0.26	0.52 ± 0.26
	48 h	1.56 ± 0.60	2.18 ± 0.09	2.08 ± 0.19	0.42 ± 0.08	3.98 ± 2.03	0.72 ± 0.30	0.74 ± 0.23
	120 h	1.62 ± 0.55	1.50 ± 0.14	1.56 ± 0.35	0.37 ± 0.19	5.73 ± 3.52	1.11 ± 0.48	1.13 ± 0.57

SUPPORTING INFORMATION REFERENCES

(1) Sun, Z.; Li, W.; Mellors, J. W.; Orentas, R.; Dimitrov, D. S. Construction of a Large Size Human Immunoglobulin Heavy Chain Variable (VH) Domain Library, Isolation and Characterization of Novel Human Antibody VH Domains Targeting PD-L1 and CD22. Front Immunol 2022, 13, 869825. DOI: 10.3389/fimmu.2022.869825.
(2) Sun, Z.; Chu, X.; Adams, C.; Ilina, T. V.; Vergara, S.; Chen, C.; Jelev, D.; Ishima, R.; Li, W.; Mellors, J. W.; et al. Discovery of a novel mesothelin specific human antibody VH domain. bioRxiv 2022.
(3) Vosjan, M. J.; Perk, L. R.; Visser, G. W.; Budde, M.; Jurek, P.; Kiefer, G. E.; van Dongen, G. A. Conjugation and radiolabeling of monoclonal antibodies with zirconium-89 for PET imaging using the bifunctional chelate p-isothiocyanatobenzyl-desferrioxamine. Nat Protoc 2010, 5 (4), 739-743. DOI: 10.1038/nprot.2010.13 From NLM Medline.
(4) Hamblett, K. J.; Senter, P. D.; Chace, D. F.; Sun, M. M.; Lenox, J.; Cerveny, C. G.; Kissler, K. M.; Bernhardt, S. X.; Kopcha, A. K.; Zabinski, R. F.; et al. Effects of drug loading on the antitumor activity of a monoclonal antibody drug conjugate. Clin Cancer Res 2004, 10 (20), 7063-7070. DOI: 10.1158/1078-0432.CCR-04-0789 From NLM Medline.
(5) Inoue, S.; Tsunoda, T.; Riku, M.; Ito, H.; Inoko, A.; Murakami, H.; Ebi, M.; Ogasawara, N.; Pastan, I.; Kasugai, K.; et al. Diffuse mesothelin expression leads to worse prognosis through enhanced cellular proliferation in colorectal cancer. Oncol Lett 2020, 19 (3), 1741-1750. DOI: 10.3892/ol.2020.11290 From NLM PubMed-not-MEDLINE.
(6) Nedrow, J. R.; White, A. G.; Modi, J.; Nguyen, K.; Chang, A. J.; Anderson, C. J. Positron emission tomographic imaging of copper 64- and gallium 68-labeled chelator conjugates of the somatostatin agonist tyr3-octreotate. Mol Imaging 2014, 13. DOI: 10.2310/7290.2014.00020 From NLM Medline.
(7) Miller, B. W.; Frost, S. H.; Frayo, S. L.; Kenoyer, A. L.; Santos, E.; Jones, J. C.; Green, D. J.; Hamlin, D. K.; Wilbur, D. S.; Fisher, D. R.; et al. Quantitative single-particle digital autoradiography with alpha-particle emitters for targeted radionuclide therapy using the iQID camera. Med Phys 2015, 42 (7), 4094-4105. DOI: 10.1118/1.4921997 From NLM Medline.
